# Accumulation of Immunity in Heavy-Tailed Sexual Contact Networks Shapes Mpox Outbreak Sizes

**DOI:** 10.1093/infdis/jiad254

**Published:** 2023-07-04

**Authors:** Hiroaki Murayama, Carl A B Pearson, Sam Abbott, Fuminari Miura, Sung-mok Jung, Elizabeth Fearon, Sebastian Funk, Akira Endo

**Affiliations:** School of Medicine, International University of Health and Welfare, Narita, Japan; Department of Infectious Disease Epidemiology, London School of Hygiene & Tropical Medicine, London, United Kingdom; Centre for Mathematical Modelling of Infectious Diseases, London School of Hygiene & Tropical Medicine, London, United Kingdom; South African DSI-NRF Centre of Excellence in Epidemiological Modelling and Analysis, Stellenbosch University, Stellenbosch, Republic of South Africa; Department of Infectious Disease Epidemiology, London School of Hygiene & Tropical Medicine, London, United Kingdom; Centre for Mathematical Modelling of Infectious Diseases, London School of Hygiene & Tropical Medicine, London, United Kingdom; Centre for Infectious Disease Control, National Institute for Public Health and the Environment (RIVM), Bilthoven, the Netherlands; Center for Marine Environmental Studies, Ehime University, Ehime, Japan; Carolina Population Center, University of North Carolina at Chapel Hill, Chapel Hill, North Carolina, USA; Centre for Mathematical Modelling of Infectious Diseases, London School of Hygiene & Tropical Medicine, London, United Kingdom; Department of Global Health and Development, London School of Hygiene & Tropical Medicine; Institute for Global Health, University College London, London, United Kingdom; Department of Infectious Disease Epidemiology, London School of Hygiene & Tropical Medicine, London, United Kingdom; Centre for Mathematical Modelling of Infectious Diseases, London School of Hygiene & Tropical Medicine, London, United Kingdom; Department of Infectious Disease Epidemiology, London School of Hygiene & Tropical Medicine, London, United Kingdom; Centre for Mathematical Modelling of Infectious Diseases, London School of Hygiene & Tropical Medicine, London, United Kingdom; School of Tropical Medicine and Global Health, Nagasaki University, Nagasaki, Japan

**Keywords:** depletion of susceptibles, heavy-tailed network, herd immunity, men who have sex with men, mpox

## Abstract

Many countries affected by the global outbreak of mpox in 2022 have observed a decline in cases. Our mathematical model accounting for heavy-tailed sexual partnership distributions suggests that mpox epidemics can hit the infection-derived herd immunity threshold and begin to decline, with <1% of sexually active men who have sex with men infected regardless of interventions or behavioral changes. We consistently found that many countries and US states experienced an epidemic peak, with cumulative cases of around 0.1% to 0.5% among men who have sex with men. The observed decline in cases may not necessarily be attributable to interventions or behavioral changes primarily.

## Teaser

Accumulation of infection-derived immunity in highly heterogeneous sexual contact networks among men who have sex with men could plausibly explain the observed decline in mpox cases in 2022 without needing to assume impacts of interventions or behavioral changes.

Since May 2022, sustained local transmission of mpox (formerly monkeypox) has been confirmed in Europe, the Americas, and other regions where the virus was not observed to circulate previously. Unlike previous outbreaks, this mpox outbreak has a novel profile—specifically, its rapid spread predominantly among men who have sex with men (MSM). This novelty can be explained by sexually associated transmission and a heavy-tailed empirical distribution of sexual partners among MSM (ie, a small number of people have disproportionately many partners), which could lead to sustained human-to-human transmission in this population but not others [1]. However, by the end of 2022, many of those countries saw an apparent slowdown in growth of cases, followed by a decline. In this study, we show that accumulation of immunity among individuals with the highest numbers of partners can explain this decline and thus should be accounted for when attempting to estimate the impact of interventions and behavioral changes.

## Methods

We developed a mathematical model of mpox transmission that accounts for the accumulation of infection-derived immunity in a heavy-tailed MSM sexual contact network (see Supplementary Materials for methodological details). We represented the heavy-tailed distribution of sexual partners among MSM over the infectious period of mpox (assumed to be 14 days) as a left-truncated Weibull distribution parameterized in our previous study [1]. We assumed that non-MSM transmission dynamics is negligible because transmission over MSM sexual networks could well approximate the overall dynamics of mpox in the current outbreak. The risk of an individual being in contact with an infectious sexual partner was modeled as being proportional to the number of sexual partners over 14 days. Upon recovery, infected individuals were assumed to develop long-term immunity and maintain their sexual behavior without further risk of reinfection. To improve robustness of the model to uncertainties in time-related parameters such as generation time and reporting delay, we used cumulative incidence as a measure of epidemic progression instead of time—that is, we directly modeled the relationship between the cumulative number of cases per MSM population and the effective reproduction number, $R_{\mathrm{eff}}$.

To compare our model outputs with observed mpox outbreak data [2, 3], we identified the period during which reported cases likely peaked in different populations (European countries, the US, Canada, and US states). We fitted Gompertz curves to the cumulative case count over time in each country and US state and estimated the cumulative number of mpox cases per MSM population size by the apparent epidemic peak (cumulative incidence proportion at a peak of an epidemic [CIPP]), where the estimated daily epidemic growth rate is consistent with a near-zero value (ie, within ±0.01). We defined the “consensus range” as a set of values that lies within the CIPPs of at least 50% of countries/states. That is, the consensus range represents the CIPP values shared by the majority of the countries/states.

## Results

**Figure 1.**
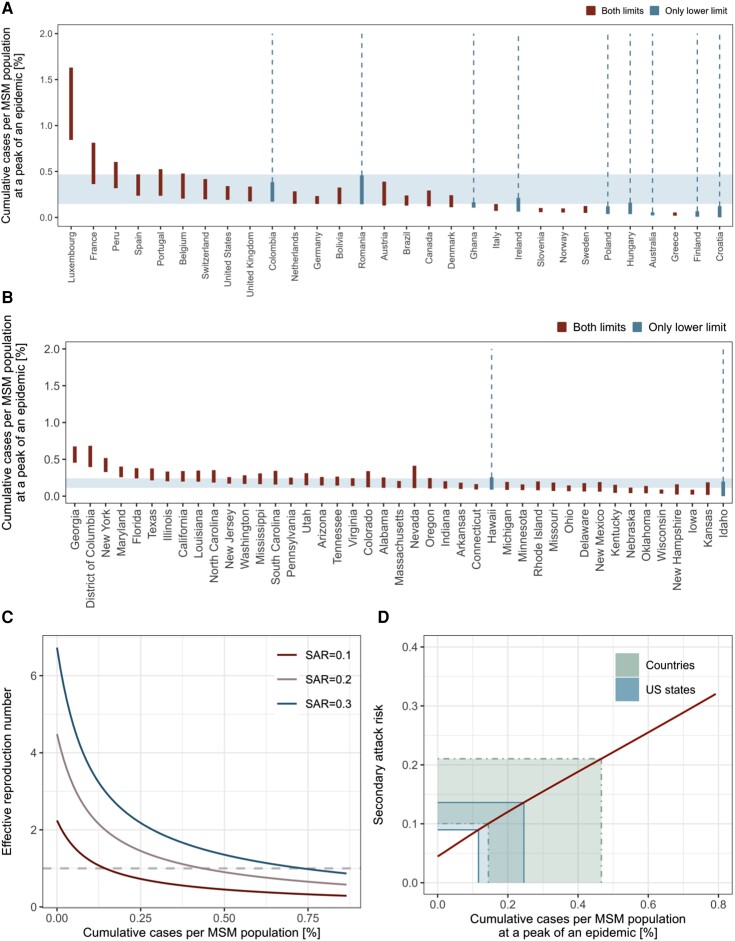
The observed and modeled number of cumulative mpox cases per MSM population. Estimated CIPP range by (*A*) country and (*B*) US state. We fitted Gompertz curves to the cumulative reported case count over time in each country and US state and estimated the cumulative number of mpox cases per MSM population size by the apparent epidemic peak, where the estimated daily epidemic growth rate is consistent with a near-zero value (ie, within ±0.01). Some countries or US states have not clearly passed the peak as of available data (last updated 15 October 2022 for countries and 15 March 2023 for US states); therefore, the upper CIPP limit is undetermined (blue bars connecting to dotted lines). Others have apparently passed the peak and have both CIPP limits (red bars). The consensus range of CIPP (values consistent with at least 50% of included countries/states) is shown with light blue shades. *C*, Modeled trajectory of the effective reproduction number ($R_{\mathrm{eff}}$) over the course of an epidemic. The reproduction number was computed for 3 possible values of SAR (0.1, 0.2, and 0.3, from bottom to top). *D*, Estimated relationship between CIPP and SAR. Thick (narrower) and thin green (wider) areas represent the global and US consensus ranges of CIPPs, respectively. CIPP, cumulative incidence proportion at a peak of an epidemic; MSM, men who have sex with men; SAR, secondary attack risk.

Using publicly available mpox outbreak data [2, 3], we identified the period during which reported cases likely peaked in different populations (European countries, the United States, Canada, and US states) and estimated the peak level per MSM population size. The consensus range among the countries suggested that their epicurves were generally consistent (though with some apparent outliers) with a saturation of growth when the cumulative case count reached 0.15% to 0.47% of the estimated MSM population size (Figure 1*A*). Moreover, 22 (73%) of 30 countries had their CIPP ranges overlapping at 0.24% (Supplementary Figure 5*A*). The consensus range among US states was 0.11% to 0.24%, and CIPPs of 25 (58%) of 43 states shared 0.16% to 0.18% in common (Figure 1*B*, Supplementary Figure 5*B*).

**Figure 2.**
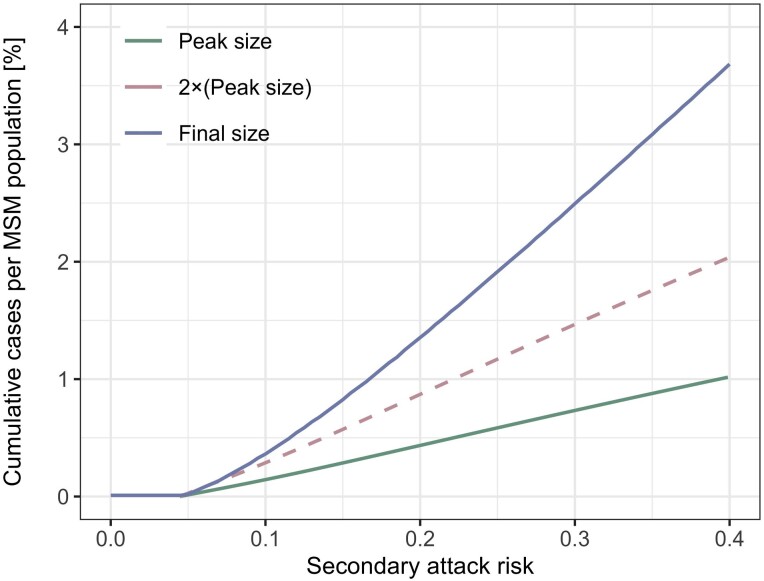
Estimated peak and final sizes by secondary attack risk in the absence of effective interventions or behavioral changes. Cumulative numbers of cases per MSM population at the peak and end of an epidemic are shown, as estimated by our model accounting for heavy-tailed sexual partnership distribution and infection-derived immunity. For comparison, a dotted line representing double the outbreak size at the peak is also included. MSM, men who have sex with men.

As individuals with the highest numbers of partners are most likely to be infected in the earliest phase of an epidemic, the effective reproduction number $R_{\mathrm{eff}}$ would rapidly decline as transmission progresses, even in the absence of any interventions or behavioral changes. Based on assumed secondary attack risk (SAR; risk of transmission per contact) values of 10%, 20%, and 30%, our model found that while $R_{0}$ (the initial value of $R_{\mathrm{eff}}$) is well above 1, $R_{\mathrm{eff}}$ rapidly decreases and crosses 1 after relatively few cases (<1% of the MSM population; Figure 1*C*). The herd immunity thresholds given an SAR of 10%, 20%, and 30% were estimated to be 0.15%, 0.43%, and 0.74% of the MSM population, respectively. These thresholds are substantially lower than the classical herd immunity threshold in a homogeneous population, $1-\frac{1}{R_{0}}$ (55%, 78%, and 85%, based on the values of $R_{0}$ in our model), and roughly align with estimated CIPP ranges. We show in Figure 1*D* that the observed consensus ranges of CIPPs are consistent with SARs of around 10% to 20% (global) or 10% to 15% (US states) if they are formed primarily by infection-derived immunity and our model assumptions are valid. We also estimated the final size of an epidemic driven by infection-derived immunity alone corresponding to different SAR values (Figure 2). The estimated final epidemic size was generally more than double the size of CIPP, contrary to outcomes for a conventional homogeneously mixing transmission model (Supplementary Materials). This suggests that the decreasing phase of an epidemic with highly heterogeneous transmission patterns may be more gradual than that of a homogeneous epidemic. The estimated final size relative to CIPP increased with SAR.

## Discussion

Many countries saw a dramatic decrease in mpox cases to which various reactions since the identification of the current outbreak could have contributed, including public health interventions such as contact tracing and vaccination [4, 5] and heightened awareness triggering behavioral changes among high-risk populations [6]. However, available evidence is overall insufficient to quantify the relative contribution of these responses to the decline in different countries, and operational indicators suggest that the impact may have been blunted by practical factors. In some settings, contact tracing and ring vaccination have been hampered by difficulty identifying contacts and a limited consent rate for postexposure vaccination among those traced [5]. Vaccine supplies were initially limited, slowing rollout of mass vaccination and precluding many countries from achieving substantial coverage before observing a peak [7, 8]—moreover, the time required for eligible individuals to complete the dosing schedule (eg, 2 doses 4 weeks apart for JYNNEOS vaccine in the United States [9]) and for immunity to be established (suggested to be up to 2 weeks by public authorities, although evidence remains limited [10]) renders prompt epidemic control by vaccination more challenging. Providing a coherent explanation to the observed decline in growth in many affected countries at different times and outbreak sizes is not straightforward.

Another key mechanism that can shape epidemic trends is the accumulation of infection-derived immunity, known as “depletion of susceptibles’ or “herd immunity” [11]. Highly heterogeneous contact patterns are known to lead to a high basic reproduction number ($R_{0}$) but lower the herd immunity threshold for immunizing infections [12]—for instance, when a small fraction of individuals exhibits disproportionately high contact rates, the initial epidemic growth could be accelerated by transmission among them, but this growth would also be short-lived as they become rapidly infected and immune and no longer contribute to the outbreak. The heavy-tailed nature of the sexual partnership distribution among MSM could create these conditions and thus explain the initial growth of mpox cases in many affected countries [1] but also their quick saturation. Britton et al [13] showed in their illustrative example that introducing heterogeneity into a SARS-CoV-2 transmission model lowers the herd immunity threshold from 67% to 50%. However, when compared with the context of respiratory infections, heterogeneity relevant to the transmission dynamics of mpox is more extreme due to the heavy-tailed nature of sexual contact patterns. As a result, our model—assuming accumulation of infection-derived immunity but no interventions or behavioral changes—replicated mpox epidemics over an MSM sexual contact network that started to decline even before 1% of MSM population experienced infection.

Attributing the observed decline in cases to interventions or behavioral changes without accounting for rapid accumulation of infection-derived immunity can bring a risk of misleading policy assessment. While these factors may also have had effects, our analyses without assuming them find plausible scenarios in which infection-derived immunity alone could explain the observed peak sizes. The observed CIPPs in the global outbreak in 2022, ranging from 0.1% to 0.5% as opposed to the classical herd immunity threshold >50% for an $R_{0}$ >2, underscore the role of heavy-tailed sexual contact networks. Our model suggests that accumulation of infection-derived immunity among high-contact individuals in those networks is likely to have played a key role in limiting peak outbreak sizes. Meanwhile, we observed variations in CIPPs that may reflect other factors, including interventions, behavioral changes, and differences in case ascertainment. Although we did not find a clear correlation between CIPPs and allocated vaccine doses in US states, we found that later epidemic onset was associated with a lower CIPP (Supplementary Figure 2). This may indicate possible impacts of interventions and behavioral changes because places with later epidemic onset may have had more lead time to implement these early in their outbreaks. However, interpreting these observed correlations requires caution because of possible confounding—states with more cases may be more likely to be allocated more vaccines, while countries and states with more active MSM populations may have been more likely to see mpox cases in the earlier phase of the outbreak. More direct and robust evidence would be required to draw conclusions regarding the effects of interventions and behavioral changes in lowering epidemic peaks. Even given a role for accumulation of infection-derived immunity in reaching epidemic turnover, it is still essential to characterize the influence of behavioral changes and public health interventions. Our model projected that in the absence of behavioral changes and interventions, the declining phase of an epidemic in a heavy-tailed contact network may be gradual, especially if the SAR is high. This means that, regardless of the factors driving peak incidence, promoting and providing effective and sustainable means of prevention, particularly vaccination, to those at risk—not only in newly affected countries but also in countries where mpox has long been endemic—serves as key operations to bring the disease spread under control and minimize the disease burden. Ensuring access to prevention for individuals at the center of sexual networks is crucial as the acceptance and effectiveness among this group would contribute most to epidemic control. This is emphasized by the severe forms of mpox among cases with advanced HIV infection [14] because the sexual network core groups and people living with HIV often overlap [15]. Sustained resourcing despite the declining trend is particularly important given that there might be (1) waning of immunity or incomplete protection or (2) turnover in the population of MSM with the most partners, which would lead to the replenishment of susceptible individuals and, therefore, epidemic potential.

Our simulations suggest that accumulation of infection-derived immunity can plausibly reproduce the observed decline in mpox cases but with a number of key limitations, especially uncertainty in characterizing the transmission network and SARs (see Supplementary Materials for details). Our model provides a parsimonious explanation of the observed decline in mpox cases, but future work with more detailed data may discriminate the role of interventions and behavioral change from saturation of infection. Such future work would help better understand the determinants of mpox epidemic trends and assess the risk of future resurgence.

## Supplementary Data

Supplementary materials are available at *The Journal of Infectious Diseases* online. Consisting of data provided by the authors to benefit the reader, the posted materials are not copyedited and are the sole responsibility of the authors, so questions or comments should be addressed to the corresponding author.

## Supplementary Material

jiad254_Supplementary_Data

## Notes

***Author contributions.*** A. E. conceived the study. H. M., F. M., S. J., and A. E. collected the data. H. M. and A. E. developed the mathematical model. H. M., C. A. B. P., and A. E. designed the study. H. M. implemented the methods. H. M. and A. E. wrote the first draft of the manuscript with feedback from C. A. B. P., S. A., F. M., S. J., E. F., and S. F. All authors read and approved the final version of the manuscript.

***Financial support*.** A. E. is supported by Japan Society for the Promotion of Science (JSPS) Overseas Research Fellowships. A. E. and F. M. are supported by the JSPS Grants-in-Aid *KAKENHI* (to A. E., 22K17329; to F. M., 20J00793). A. E. and H. M. are supported by the foundation for the Fusion of Science and Technology (to A. E.) and the Japan Science and Technology Agency Precursory Research for Embryonic Science and Technology (to A. E., JPMJPR22R3). S. F. and S. A. are supported by Wellcome Trust (to S. F., 210758/Z/18/Z). C. A. B. P. is supported by the Innovative Medicines Initiative 2 joint undertaking under grant agreement EBOVAC3 (800176); this joint undertaking receives support from the European Union’s Horizon 2020 Research and Innovation Programme and the European Federation of Pharmaceutical Industries and Associations (EFPIA). S. J. is supported by the Centers for Disease Control and Prevention SHEPheRD (200-2016-91781). E. F. is supported by the Medical Research Council, United Kingdom Research and Innovation (MR/S020462/1).

***Data and code availability.*** All data used in this study are publicly available. The analysis codes are available from a GitHub repository: https://github.com/hiroaki-murayama/MPX_depletion_susceptibles.
