# Supplementary material for: Accumulation of Immunity in Heavy-Tailed Sexual Contact Networks Shapes Mpox Outbreak Sizes
Source: J Infect Dis. 2023 Jul 4;229(1):59–63. doi: 10.1093/infdis/jiad254 (PMC10786257; doi:10.1093/infdis/jiad254)
Supplement: jiad254_Supplementary_Data [file jiad254_supplementary_data.pdf]

Supplementary Material: Accumulation of immunity in heavy-tailed sexual contact networks shapes mpox outbreak sizes

Authors: Hiroaki Murayama[a], Carl A. B. Pearson[b,c,d], Sam Abbott[b,c], Fuminari Miura[e,f], Sung-mok Jung[g], Elizabeth Fearon[c,h,i], Sebastian Funk[b,c], Akira Endo[b,c,j,1]

Affiliations:
a. School of Medicine, International University of Health and Welfare, Narita, Japan
b. Department of Infectious Disease Epidemiology, London School of Hygiene & Tropical Medicine, London, UK
c. Centre for Mathematical Modelling of Infectious Diseases, London School of Hygiene & Tropical Medicine, London, UK
d. South African DSI-NRF Centre of Excellence in Epidemiological Modelling and Analysis (SACEMA), Stellenbosch University, Stellenbosch, Republic of South Africa
e. Centre for Infectious Disease Control, National Institute for Public Health and the Environment (RIVM), Bilthoven, the Netherlands
f. Center for Marine Environmental Studies (CMES), Ehime University, Ehime, Japan
g. Carolina Population Center, University of North Carolina at Chapel Hill, Chapel Hill, NC, USA
h. Department of Global Health and Development, London School of Hygiene & Tropical Medicine, London, UK
i. Institute for Global Health, University College London, London, UK
j. School of Tropical Medicine and Global Health, Nagasaki University, Nagasaki, Japan

1. **Corresponding author**: Akira Endo. Email: akira.endo@lshtm.ac.uk.

Materials and Methods

Data source

We used the cumulative incidence data of reported mpox cases in countries and US states that have ever observed at least 10 cases over the successive 5 days as of 15 October 2022 for countries and 15 March 2023 for US states (1,2). Due to the data availability of MSM population size and our minimum threshold for mpox case counts for stable estimation, we limited our analysis of CIPP to the countries listed in Table S1 and US states. As the cumulative case counts in US states are continuously updated on the CDC website, we referred to a public repository and an internet archive for historical data from CDC as necessary (3,4). The total and estimated MSM population sizes in included countries and US states were collected from previous studies and public data sources (5–9). We combined two data sources on country-level MSM population sizes for our main analysis (5,9). If a country is included in both of the sources, we used the more recent figure (Table S1). As a sensitivity analysis, we also present the results using a single data source as of 2009 (5) (Figure S6). The MSM population was defined as “the population which contributes to the HIV epidemic among MSM” in (5) and “the number of men who had sex with men within the past 5 years” in (6), respectively. The Joint United Nations Programme on HIV/AIDS (UNAIDS) data (9) consists of a collection of data sources, most of which used the presence of male-to-male sexual act over a specific time period to define MSM (10). We assumed that these data approximately represent the sexually active MSM population considered in our model. We also used the data of allocated doses of vaccine in US states as of 15 September 2022 (11). Similar data on vaccination across countries was not available.

Table S1. Country-specific MSM population size estimates.

Country	MSM population size	Year	Reference
Australia	263,500	2015	(9)
Austria	70,985	2009	(5)
Belgium	106,336	2009	(5)
Bolivia	35,500	2021	(9)
Brazil	2,000,000	2013	(9)
Canada	349,800	2014	(9)
Colombia	356,700	2021	(9)
Croatia	29,500	2013	(9)
Denmark	50,000	2013	(9)
Finland	53,118	2009	(5)
France	330,000	2011	(9)
Germany	750,000	2013	(9)
Ghana	54,800	2017	(9)
Greece	94,000	2015	(9)
Hungary	53,404	2009	(5)
Ireland	86,500	2015	(9)
Italy	359,315	2009	(5)
Luxembourg	1,749	2009	(5)
Netherlands	230,000	2013	(9)
Norway	47,483	2009	(5)
Peru	260,000	2019	(9)
Poland	134,981	2009	(5)
Portugal	107,328	2009	(5)
Romania	10,500	2011	(9)
Slovenia	21,591	2009	(5)
Spain	890,200	2015	(9)
Sweden	100,000	2013	(9)
Switzerland	80,000	2011	(9)
United Kingdom	613,658	2009	(5)
United States	4,500,000	2013	(9)

Estimation of peak size

To identify the period when an epidemic was most likely reaching its peak, we fitted a Gompertz curve to cumulative incidence in each of the included countries and US states, which serves as an approximation of saturating epidemic growth (12,13). Let $F(t)$ be the Gompertz function:

$$F(t) = K\exp\left(\log\left(\frac{F_0}{K}\right)\exp\left(-at\right)\right), \quad (1)$$

where a (decay rate of the growth), F_0 (initial number of cases) and K (carrying capacity) are the parameters that characterise the Gompertz function.

We defined the local growth rate of the Gompertz curve at time *t* as:

$$\phi(t) = \frac{\frac{d^2F(t)}{dt^2}}{\frac{dF(t)}{dt}} = a\left(\log\left(\frac{K}{F_0}\right)\exp(-at) - 1\right), \tag{2}$$

Note that, because $\frac{dF(t)}{dt} = f_0 \exp\big(\phi(t)\big)$ satisfy the condition $\phi(t) = \frac{\frac{d^2F(t)}{dt^2}}{\frac{dF(t)}{dt}}$, $\phi(t)$ can be interpreted as the growth rate of an exponential function that locally approximates the epidemic curve. The peak of an epidemic is characterised as the point where the local growth rate (ϕ) is zero: around this point the cumulative incidence is expected to be approximately linear. We therefore considered the period during which the estimated ϕ is sufficiently close to zero ($|\phi| \leq 0.01$) as the possible time range for the epidemic peak. We assumed that the observed cumulative incidence follows the Gompertz function with normally-distributed errors, i.e. $\mathrm{Normal}(\mathrm{mean} = F(t), \mathrm{sd} = \sigma)$. We estimated a, F_0, K, and σ using the Markov-chain Monte Carlo (MCMC) method (via the {rstan} package in R (14)). We employed an improper flat prior ($\mathrm{Uniform}(0, \infty)$) for σ and weakly-informative priors for a, F_0 and K: $\mathrm{HalfNormal}(0, 1)$ for a (given that it would be below 1), $\mathrm{LeftTruncatedNormal}(10, 50)$ for F_0 truncated at 0 (reflecting that all the included countries/US states hold at least 10 cases) and $\mathrm{HalfNormal}(0, 0.025N_c)$ for K, where N_c is the total population size of country c. The prior for K reflects that carrying capacity would be at least under 2.5% of the total population size, given that the relative MSM population size is reported to be up to 5.6% of the adult male population size in Europe (5). We obtained 10,000 MCMC samples from four chains using the Hamiltonian Monte Carlo algorithm with No-U-Turn-Sampler, where the first 2,000 warm-up iterations were discarded. The resulting MCMC samples showed an R-hat statistic of below 1.05 and an effective sample size of at least 200. We identified the period where the 95% credible interval of estimated local growth rate contains a value within the range $|\phi| \leq 0.01$ and defined CIPP as the cumulative number of cases per MSM population size during this period (the upper limit of CIPP may be undefined if the epidemic has not yet clearly passed the peak). We then constructed a consensus range of CIPP across countries ("global consensus range") and US states ("US consensus range") included in the analysis. We defined the consensus range as a set of values that lie within the CIPP of at least 50% of included countries/states. That is, any value within the consensus range is consistent with the majority of the country/state CIPPs.

Epidemic model

To describe the heterogeneity of sexual contact networks among MSM, we used a left-truncated Weibull distribution, $w(x)$, estimated to represent the number of sexual partners over the infectious period of mpox elsewhere (15). In the original study, the distribution was fitted to the annual sexual partnership data for MSM in the UK and then rescaled to the assumed infectious period of mpox of 21 days. The left-truncated Weibull distribution is defined as

$$w(x) = \frac{\alpha}{\theta}\left(\frac{x}{\theta}\right)^{\alpha-1}\exp\left(\left(\frac{1}{\theta}\right)^{\alpha} - \left(\frac{x}{\theta}\right)^{\alpha}\right), \tag{3}$$

where α and θ represent the shape and the scale parameters ($\alpha, \theta > 0$), respectively. A Weibull distribution with a shape $0<\alpha<1$ has a heavy tail (16) and has been shown to accurately represent the empirical sexual partnership distribution among MSM (15), where a small proportion of individuals have disproportionately many partners. In the present study we instead assumed 14 days to reflect updated epidemiological knowledge (17,18). The resulting Weibull distribution of the mean number of partners over 14 days has a shape parameter $\alpha = 0.10$ and a scale parameter $\theta = 5.2\times10^{-11}$. Alternatively, the distribution is characterised by a Pareto-approximated exponent $\kappa = 1.1$ (i.e. the exponent parameter of a Pareto distribution that approximates the body part of the Weibull distribution (15); $\kappa = \alpha/\theta^{\alpha}$) and the upper 1 percentile of 16.

We developed a dynamical model of mpox transmission that accounts for the heavy-tailed sexual partnership distribution among MSM. Instead of calendar time, we use the cumulative force of infection (CFOI) to measure the progression of an epidemic for mathematical convenience. Let $S(\Lambda, x)$ be the population fraction of susceptible individuals with degree x when CFOI is Λ. We assumed that the population is fully susceptible at the start of an epidemic, i.e. $S(\Lambda = 0) = \int_0^\infty S(\Lambda = 0, x)\, dx = \int_0^\infty w(x)\, dx = 1$. We assumed that individuals are exposed to mpox virus at a probability proportional to their degree (i.e. those with a large number of partners are more likely to be chosen) and that infected individuals develop permanent immunity to be protected from future infections while retaining their original sexual behaviour for the rest of the epidemic. The process of depletion of susceptibles is then described as

$$\frac{\partial S(\Lambda, x)}{\partial \Lambda} = -\frac{p_x S(\Lambda, x)}{w(x)}, \tag{4}$$

where p_x is the relative frequency of x among cases ($p_x = \frac{x w(x)}{\langle x \rangle}$) and $\langle x \rangle$ is the mean degree ($\langle x \rangle = \int_0^\infty x w(x)\, dx$). This equation represents the reduction in the susceptible population with degree x associated with a small increment in CFOI. The incremental CFOI is assumed to be distributed proportionally to degree x (represented by p_x). Among the exposed individuals with degree x, only the proportion $\frac{S(\Lambda,x)}{w(x)}$ is susceptible; thus, the net reduction is $\frac{p_x S(\Lambda,x)}{w(x)}$.

Solving Equation (4), we get a closed form of $S(\Lambda, x)$ and cumulative incidence proportion $I(\Lambda)$ as:

$$S(\Lambda, x) = w(x) \exp\left(-\frac{\Lambda x}{\langle x \rangle}\right), \tag{5}$$

$$I(\Lambda) = 1 - \int_0^\infty S(\Lambda, x)\, dx. \tag{6}$$

The effective reproduction number (R_{eff}) over a sexual contact network is then derived using the mean excess degree of susceptibles. The mean excess degree of susceptibles ($\langle e_S(\Lambda)\rangle$) is given as:

$$\langle e_S(\Lambda)\rangle = \frac{\int_1^\infty x(x-1)S(\Lambda,x)dx}{\int_0^\infty xS(\Lambda,x)dx}. \tag{7}$$

Denoting the secondary attack risk (SAR) per sexual partnership as β, we obtain the following equation for R_{eff}:

$$R_{\text{eff}}(\Lambda) = \beta\langle e_S(\Lambda)\rangle \frac{\int_0^\infty xS(\Lambda,x)dx}{\langle x\rangle} = \frac{\beta\int_1^\infty x(x-1)S(\Lambda,x)dx}{\langle x\rangle}. \tag{8}$$

Note that when $\Lambda = 0$, i.e. at the start of an epidemic, $R_{\text{eff}}(\Lambda)$ corresponds to the basic reproduction number (R_0) as defined in (15).

The peak of an epidemic is the point where $R_{\text{eff}}(\Lambda) = 1$. By rearranging Equation (8), we get an estimator for SAR (β):

$$\beta = \frac{\langle x\rangle}{\int_1^\infty x(x-1)w(x)\ \exp\left(-\frac{\Lambda_{\text{peak}}x}{\langle x\rangle}\right)dx}, \tag{9}$$

where Λ_{peak} is the CFOI at the peak of an epidemic. We estimated SAR that renders our model consistent with the global and US consensus ranges of CIPP using Equations (6) and (9); that is, assuming that interventions or behavioural changes have negligible effects on peak sizes and that case ascertainment was sufficiently high, the estimated consensus range of CIPPs corresponds to $I(\Lambda_{\text{peak}})$, which allows us to compute β. We assumed that observed cases are predominantly among MSM who acquired infection via sexually-associated contacts (15,19) and that the transmission dynamics is thus well described by spread within a closed MSM population.

We also derived the expected final size of an epidemic using the following equation on the CFOI at the final size (Λ_{final}):

$$\int_0^{\Lambda_{\text{final}}} R_{\text{eff}}(\Lambda)\, dI(\Lambda) = I(\Lambda_{\text{final}}). \tag{10}$$

We numerically solved this equation by a grid search with a step size of 10^{-4}, where the integral was approximated by a Riemann sum with a step size of 10^{-6}.

Relationship between peak and final sizes in a homogeneously mixing population

We showed in Fig. 2 that the final size of an epidemic over a heavy-tailed sexual contact network in our model is generally larger than double the size of the peak. Here we provide a quick proof that this is contrary to the feature of a homogeneous mixing Susceptible-Infectious-Recovered (SIR) model. Let y and z represent the cumulative incidence per capita at the peak (peak size) and at the end of an epidemic (final size), respectively. These are defined by the following equations in a homogeneous mixing SIR model (20):

$$y = 1 - \frac{1}{R_0},$$

$$z = 1 - \exp\left(-R_0 z\right).$$

The ratio between the peak and final sizes is then given as $r(z) = \frac{y}{z} = \frac{1 + \frac{z}{\log(1-z)}}{z}$. We can show that $r(z)$ is a monotonically increasing function for $0 < z < 1$ as follows. The derivative of $r(z)$ is

$$\frac{dr}{dz} = \frac{1}{(1-z)(1-z)} - \frac{1}{z^2},$$

whose sign matches that of $z^2 - (1-z)(1-z)$. We get $\log(1-z) < -z < 0$, which yields

$$z^2 - (1-z)(1-z) > z^2 - (1-z) > 0.$$

With $r(z) = 0.5$, it is assured that $r > 0.5$, i.e. the final size of an epidemic is always smaller than double the peak size.

Study limitations

Our analysis holds several key limitations. MSM population size estimates used to calculate CIPP were subject to uncertainties and potential biases (5,6). For example, MSM population size estimates for multiple countries are indicated to be less reliable by the authors (5), which may have affected some of the countries that showed outlier values of CIPP, e.g. Luxemburg. We assumed that the sexual partnership distribution in UK estimated in the previous study (15) applies to countries experiencing an epidemic among MSM. Some deviations from the UK partnership distributions are expected in different settings, although our sensitivity analysis suggested the robustness of our qualitative conclusions (Fig. S3). We found possible weak positive correlations between CIPPs and MSM population sizes (Spearman's correlation 0.32 [-0.03, 0.64] among countries and 0.61 [0.38, 0.79] among US states) (Fig. S4). They may indicate variations in partnership distributions or other factors including case ascertainment between large and small countries/cities. We projected R_{eff} over time assuming SAR values of 10%, 20% and 30%, which are roughly consistent with our CIPP consensus ranges and also existing household SAR estimates of ~10% (or slightly above, reflecting the more intimate nature of sexually-associated contact). However, the actual range of SAR values in the current mpox outbreak are still scarcely known and subject to debate. One study reported a PCR positivity of 66% among sexual contacts of confirmed cases (12 out of 18) (21). However, 4 of these positive contacts were reported to have shown low viral loads along with atypical or no symptoms, and the potential for onward transmission of such cases is unclear. Since our study implicitly assumed that only infectious individuals among contacts contribute to SAR, these results may not necessarily contradict our results. Furthermore, we assumed that most infections were reported and reflected on the observed CIPPs when estimating the possible range of SAR (as shown in Fig 1D). If cases were

significantly underreported (i.e. true CIPPs were higher than observed), a higher SAR than we used would be expected. We did not consider network assortativity or clustering, which may have led to overestimation of final size (22). We also assumed that the influence of imported cases on the local dynamics at country- and US state-levels are negligible; however, this may not have been the case especially in populations with a small case count. Finally, we reiterate that our findings should not be viewed as evidence on the effects of interventions and behavioural changes in the current outbreak. Our model plausibly explained CIPPs at both country- or US state-levels that are at similar order and substantially lower than the classical herd immunity threshold without needing the effect of interventions or behavioural changes. However, such patterns could also be observed if included countries and US states exhibited similar levels of interventions or behavioural changes at time of their epidemic peaks. In that case, the SAR values consistent with our CIPP consensus ranges may already reflect these suppression effects and the true SAR might be higher. Further studies incorporating our findings on the saturation effect from infection-derived immunity will enable us to better understand the evolving situations of mpox epidemiology.

Additional Figures

Fig. S1. **Correlation between CIPP and allocated doses of vaccine by the peak among US states**. The lower limits of CIPP in US states are displayed as dots. The Spearman's correlation coefficient is 0.33 [95% confidence interval: 0.01, 0.61].

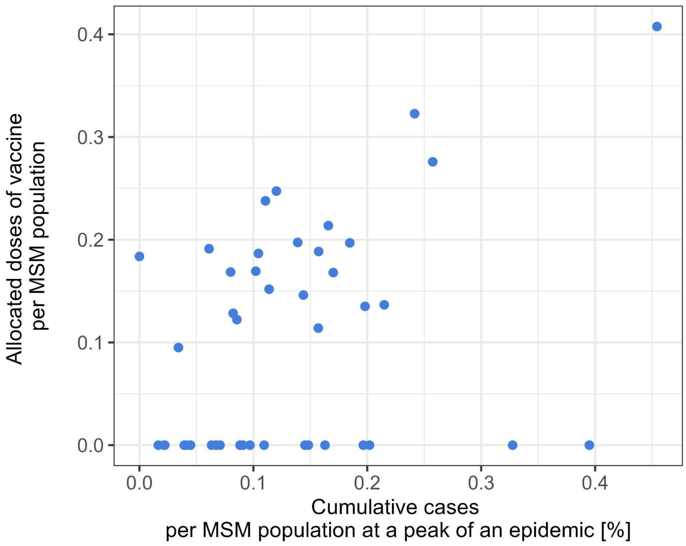

Fig. S2. **Correlation between CIPP and date of reporting the 10th case**. The lower limits of CIPP (A) by country and (B) by US-state are displayed as dots. The Spearman's correlation coefficients are -0.34 [-0.69, 0.05] (countries) and -0.77 [-0.88, -0.59] (US states).

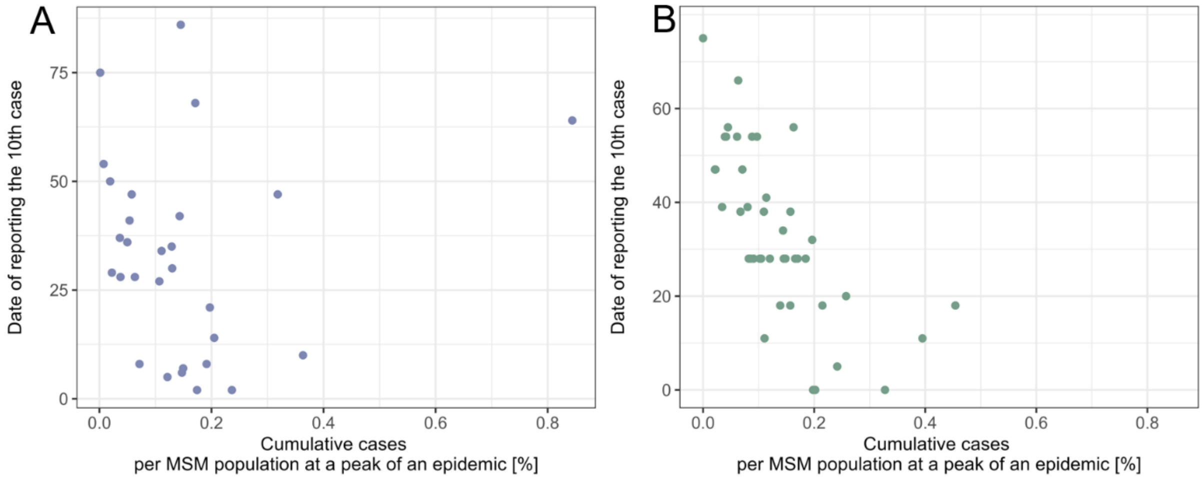

Fig. S3. **Sensitivity to the variations in the MSM sexual partnership distribution**. Each dotted line shows the relationship between CIPP and secondary attack risk reflecting possible

variations in the Weibull distribution representing the sexual partnerships among MSM. Changes to the assumed Weibull distribution was represented by a Pareto-approximated exponent κ and the upper 1st percentile. Thick and thin green areas represent the global and US consensus ranges of CIPP, respectively.

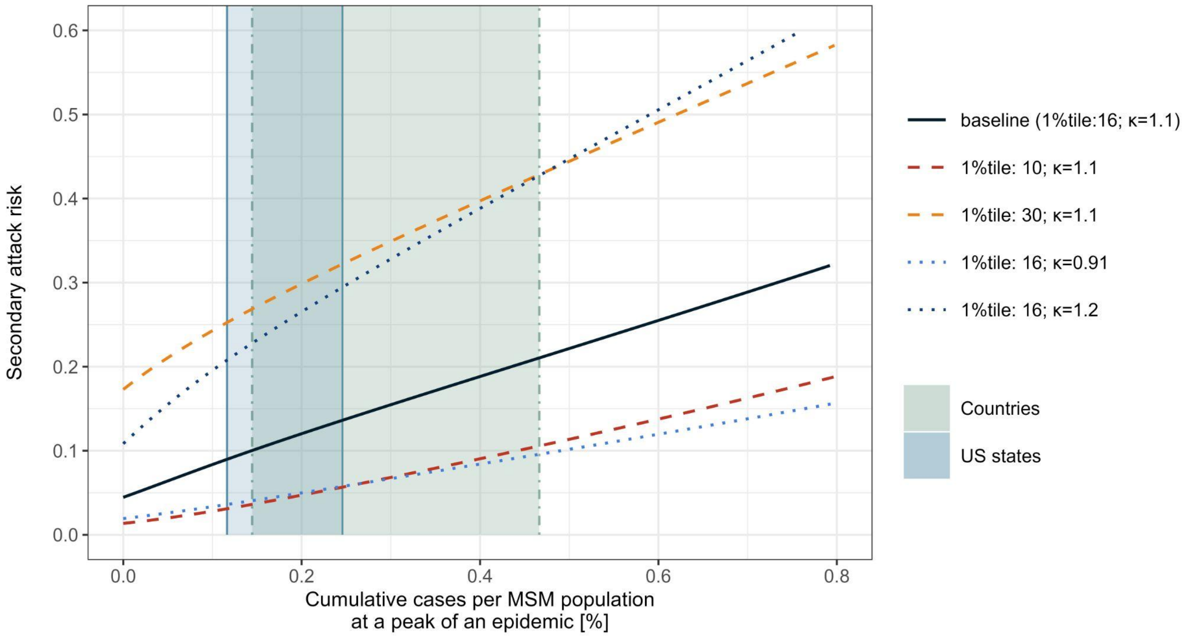

Fig. S4. **Correlation between CIPP and MSM population size**. The lower limits of CIPP (A) by country and (B) by US-state are displayed as dots. The Spearman's correlation coefficients are 0.32 [-0.03, 0.63] (countries) and 0.61 [0.38, 0.78] (US states).

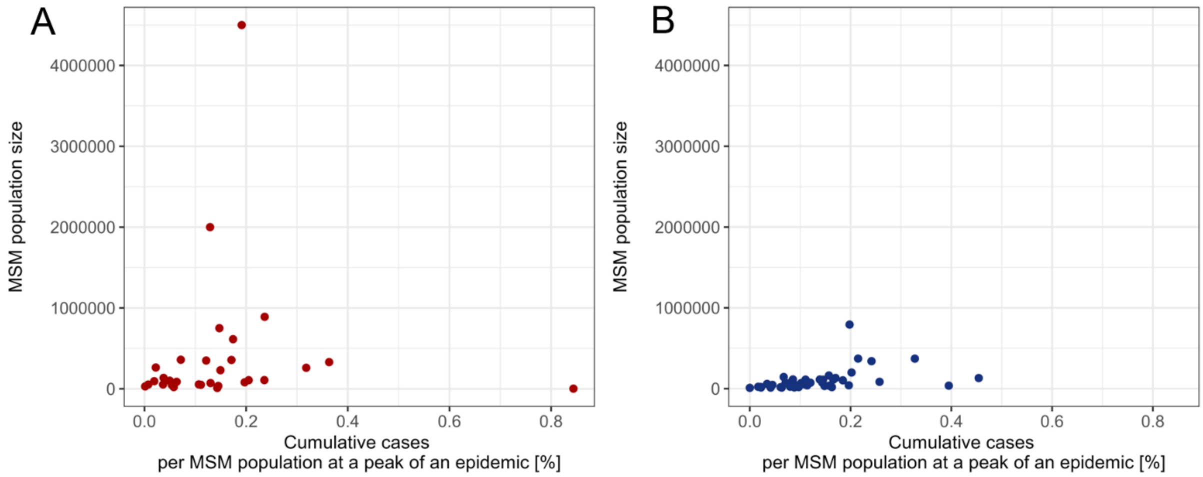

Fig. S5. **Consensus ranges of CIPP.** Red line shows the number of countries/states whose CIPP includes the specific CIPP value. The light blue shaded areas represent the consensus ranges of CIPP. Blue dot line shows the threshold of 50% used to define the consensus ranges for (A) included countries and (B) US states.

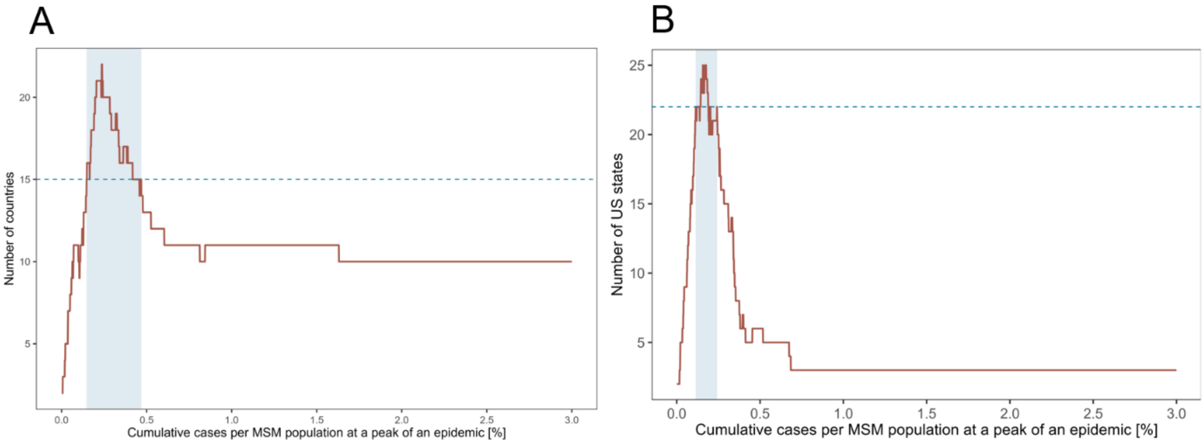

Fig. S6. Sensitivity to the MSM population sizes. CIPPs are computed relying solely upon data at country-level by Marcus et al. as of 2009. The upper limit of CIPP is represented by blue bars; others have apparently passed the peak and have both limits for CIPP (red bars). The consensus range of CIPP (values consistent with at least 50% of included countries/states) is shown with light blue shades (0.13%-0.39%). This indicates the robustness of MSM population sizes on the estimates of CIPPs and the consensus range.

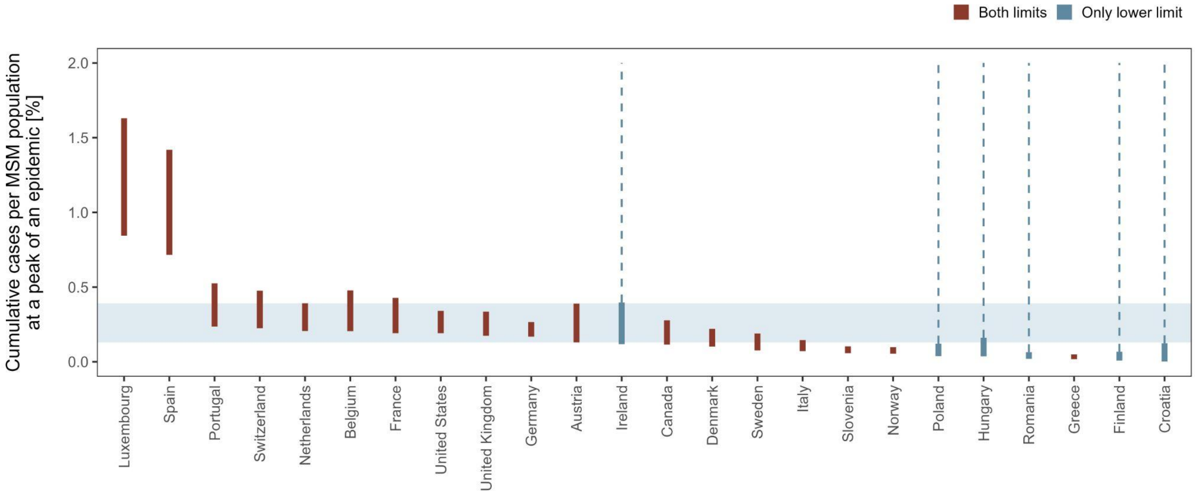

Fig. S7. **Fitted Gompertz curves and estimated local growth rates in countries and US states.** Purple bars represent the reported cumulative number of mpox cases. Thick blue lines show the median estimates of (A) epidemic curves and (B) growth rates and blue shaded areas their 95% credible intervals. Thin purple areas show the range where the growth rate takes a near-zero value (i.e. within ±0.01).

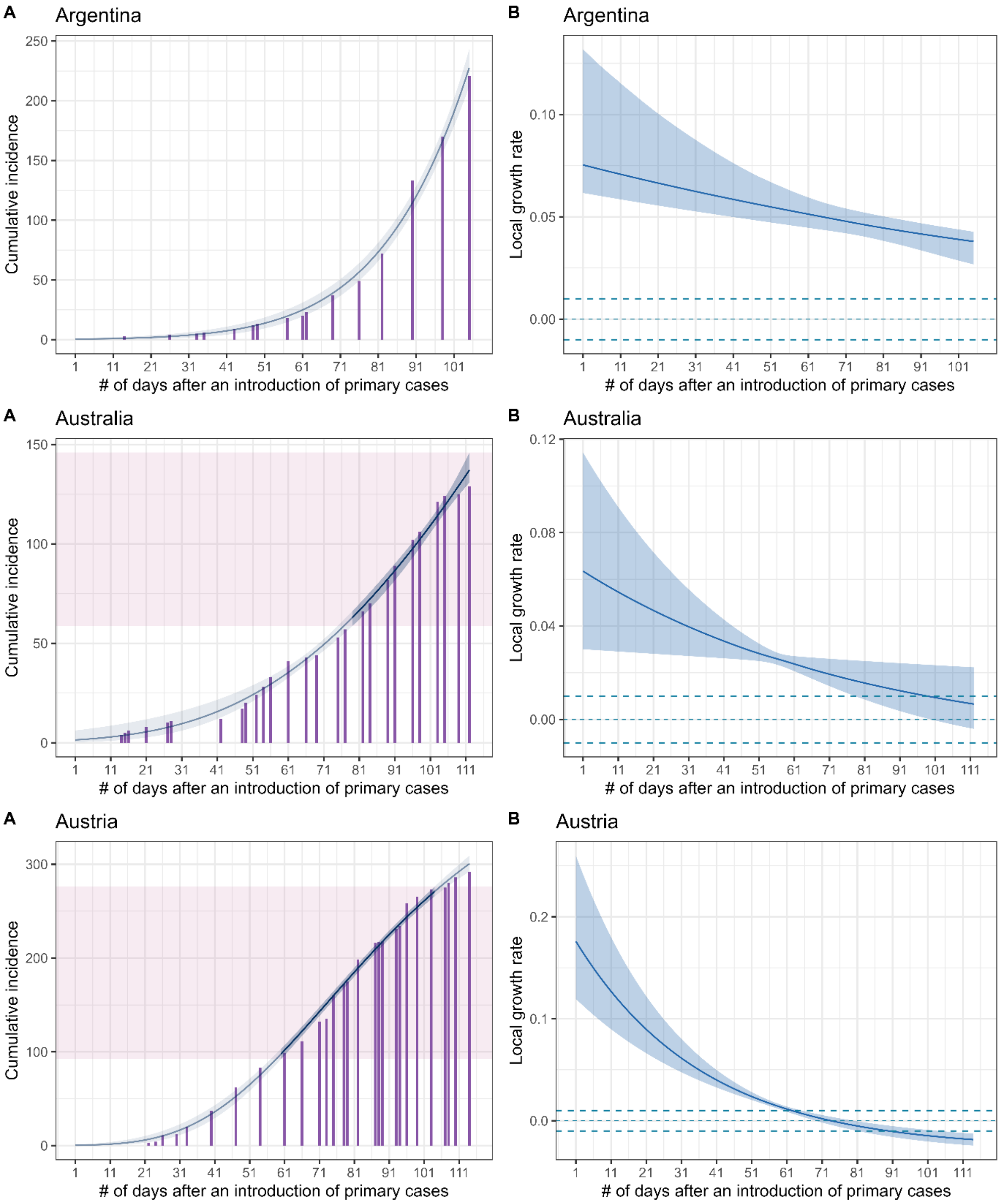

A
Argentina
Cumulative incidence
0
50
100
150
200
250
1
11
21
31
41
51
61
71
81
91
101
# of days after an introduction of primary cases
B
Argentina
Local growth rate
0.00
0.05
0.10
1
11
21
31
41
51
61
71
81
91
101
# of days after an introduction of primary cases
A
Australia
Cumulative incidence
0
50
100
150
1
11
21
31
41
51
61
71
81
91
101
111
# of days after an introduction of primary cases
B
Australia
Local growth rate
0.00
0.04
0.08
0.12
1
11
21
31
41
51
61
71
81
91
101
111
# of days after an introduction of primary cases
A
Austria
Cumulative incidence
0
100
200
300
1
11
21
31
41
51
61
71
81
91
101
111
# of days after an introduction of primary cases
B
Austria
Local growth rate
0.0
0.1
0.2
1
11
21
31
41
51
61
71
81
91
101
111
# of days after an introduction of primary cases

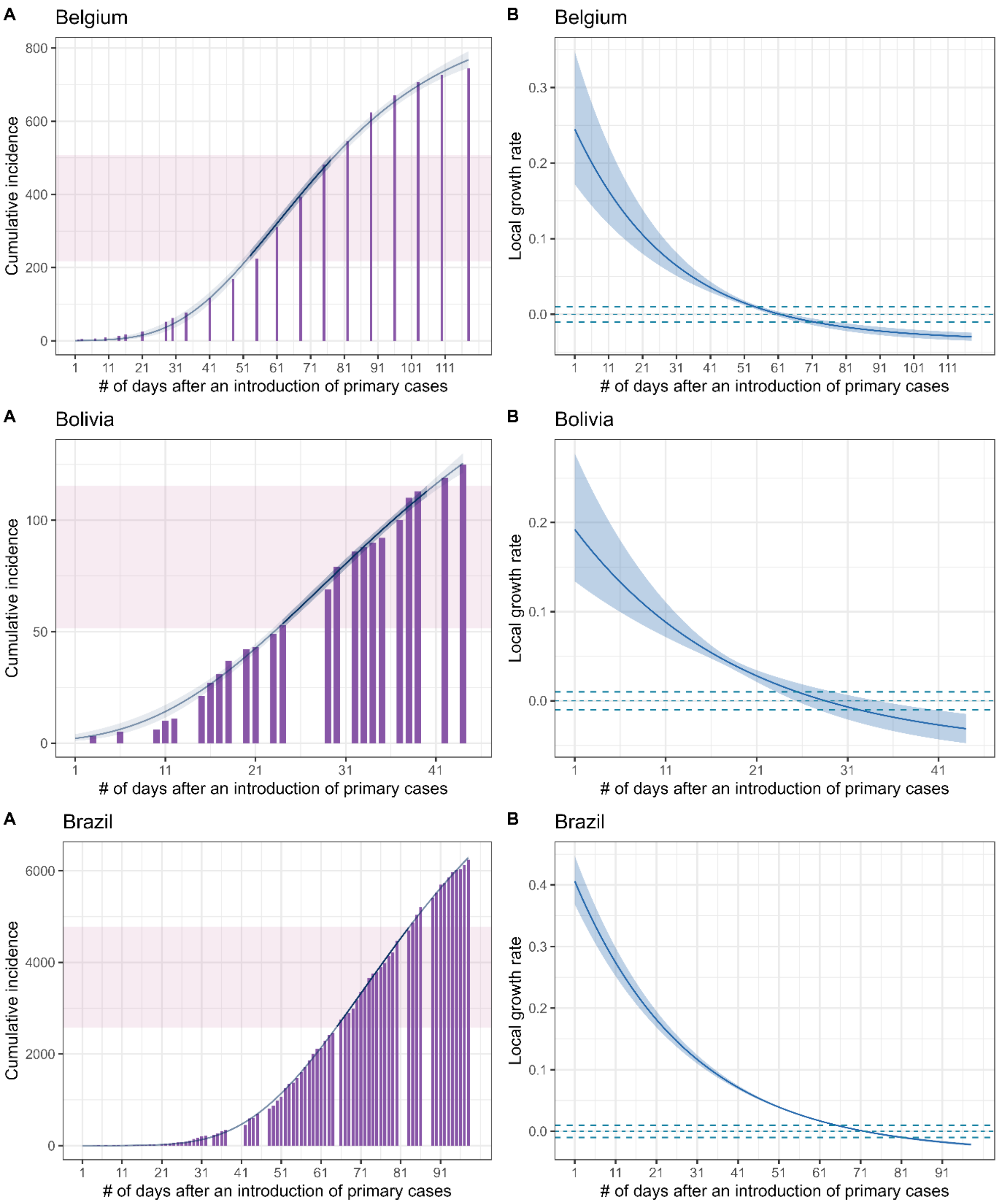
A
Belgium
Cumulative incidence
800
600
400
200
0
1 11 21 31 41 51 61 71 81 91 101 111
# of days after an introduction of primary cases
B
Belgium
Local growth rate
0.3
0.2
0.1
0.0
1 11 21 31 41 51 61 71 81 91 101 111
# of days after an introduction of primary cases
A
Bolivia
Cumulative incidence
100
50
0
1 11 21 31 41
# of days after an introduction of primary cases
B
Bolivia
Local growth rate
0.2
0.1
0.0
1 11 21 31 41
# of days after an introduction of primary cases
A
Brazil
Cumulative incidence
6000
4000
2000
0
1 11 21 31 41 51 61 71 81 91
# of days after an introduction of primary cases
B
Brazil
Local growth rate
0.4
0.3
0.2
0.1
0.0
1 11 21 31 41 51 61 71 81 91
# of days after an introduction of primary cases

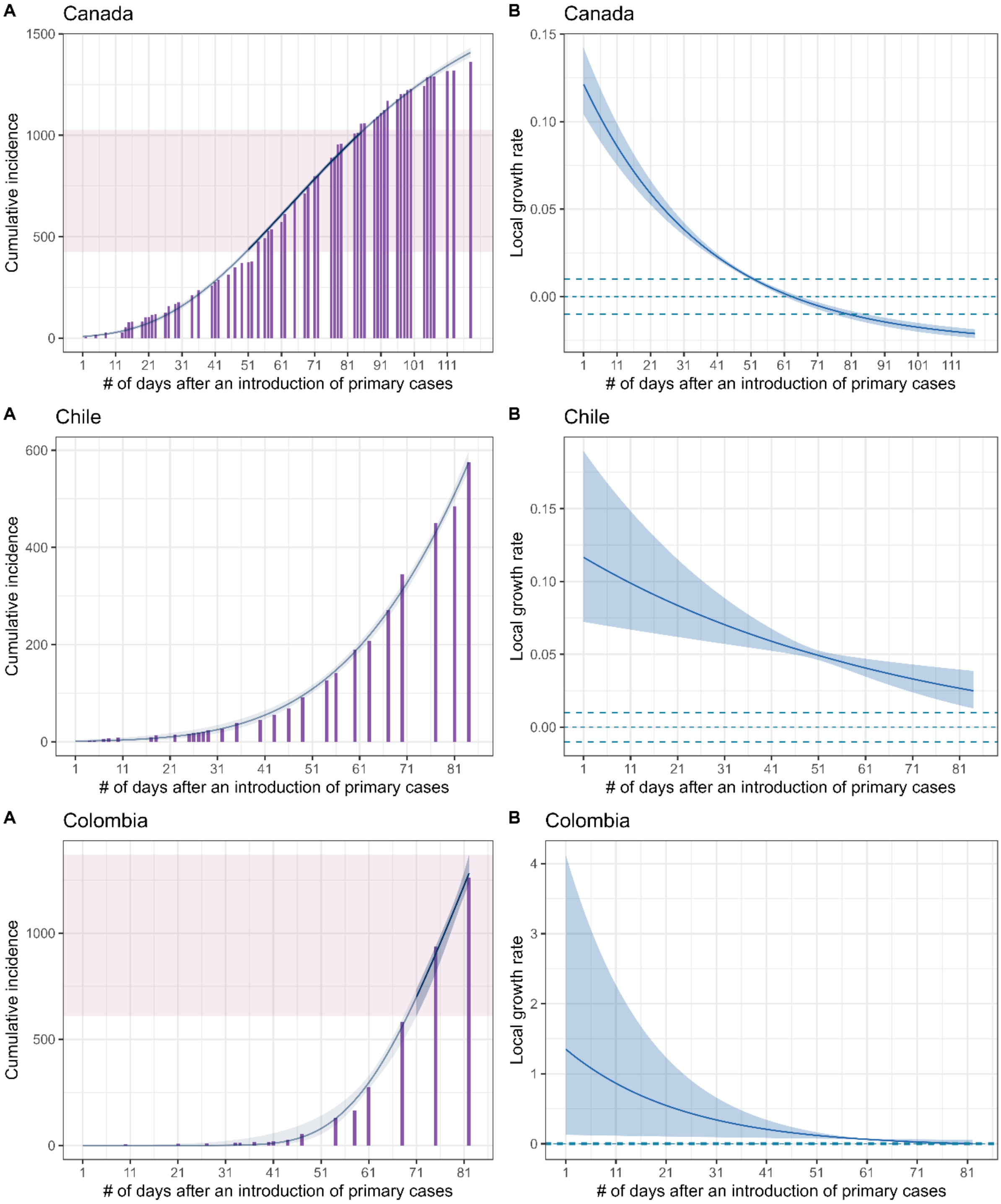

A
Canada
Cumulative incidence
1500
1000
500
0
1
11
21
31
41
51
61
71
81
91
101
111
# of days after an introduction of primary cases
B
Canada
Local growth rate
0.15
0.10
0.05
0.00
# of days after an introduction of primary cases
A
Chile
Cumulative incidence
600
400
200
0
# of days after an introduction of primary cases
B
Chile
Local growth rate
0.15
0.10
0.05
0.00
# of days after an introduction of primary cases
A
Colombia
Cumulative incidence
1000
500
0
# of days after an introduction of primary cases
B
Colombia
Local growth rate
4
3
2
1
0
# of days after an introduction of primary cases

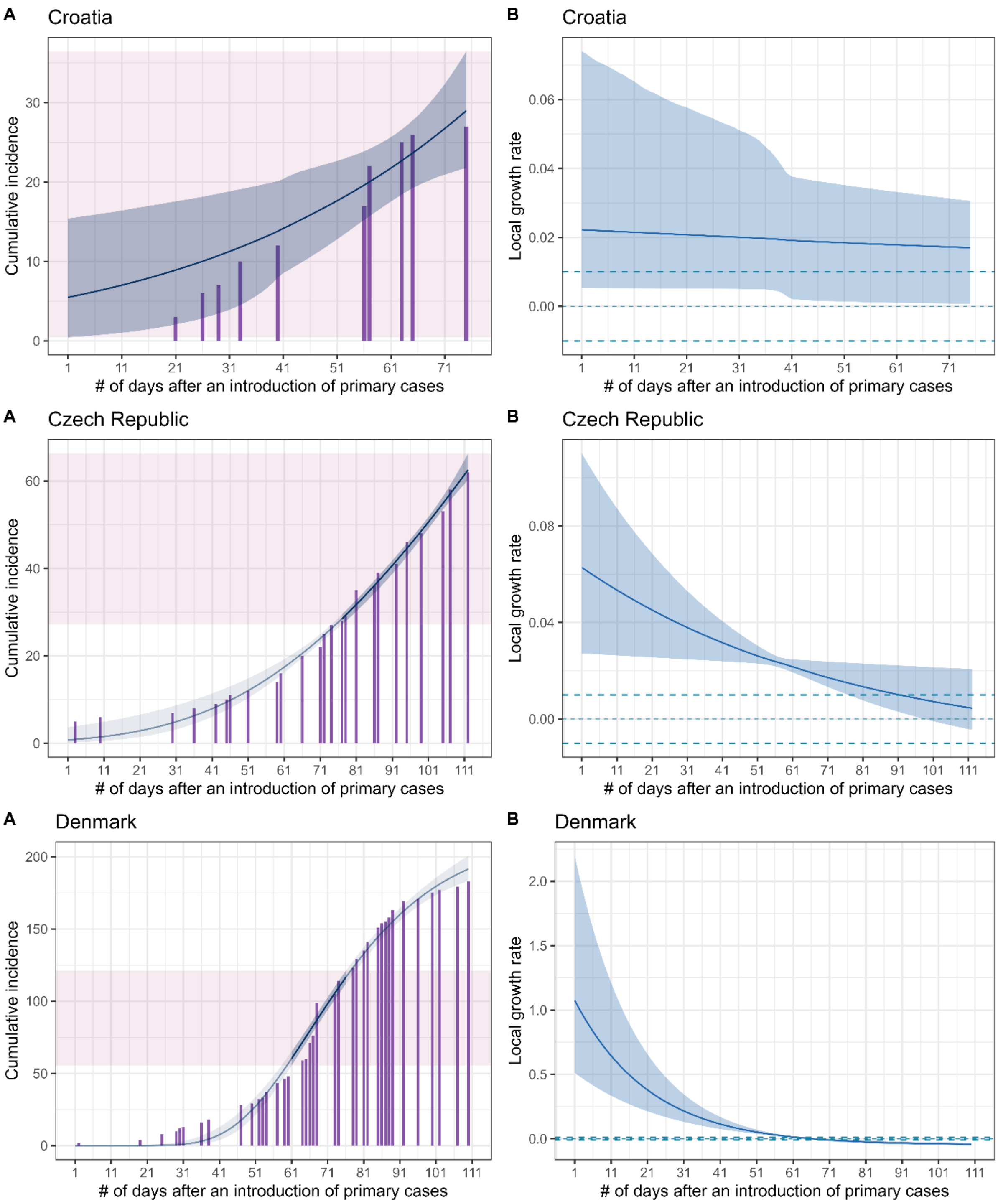
A
Croatia
Cumulative incidence
0
10
20
30
1
11
21
31
41
51
61
71
# of days after an introduction of primary cases
B
Croatia
Local growth rate
0.00
0.02
0.04
0.06
1
11
21
31
41
51
61
71
# of days after an introduction of primary cases
A
Czech Republic
Cumulative incidence
0
20
40
60
1
11
21
31
41
51
61
71
81
91
101
111
# of days after an introduction of primary cases
B
Czech Republic
Local growth rate
0.00
0.04
0.08
1
11
21
31
41
51
61
71
81
91
101
111
# of days after an introduction of primary cases
A
Denmark
Cumulative incidence
0
50
100
150
200
1
11
21
31
41
51
61
71
81
91
101
111
# of days after an introduction of primary cases
B
Denmark
Local growth rate
0.0
0.5
1.0
1.5
2.0
1
11
21
31
41
51
61
71
81
91
101
111
# of days after an introduction of primary cases

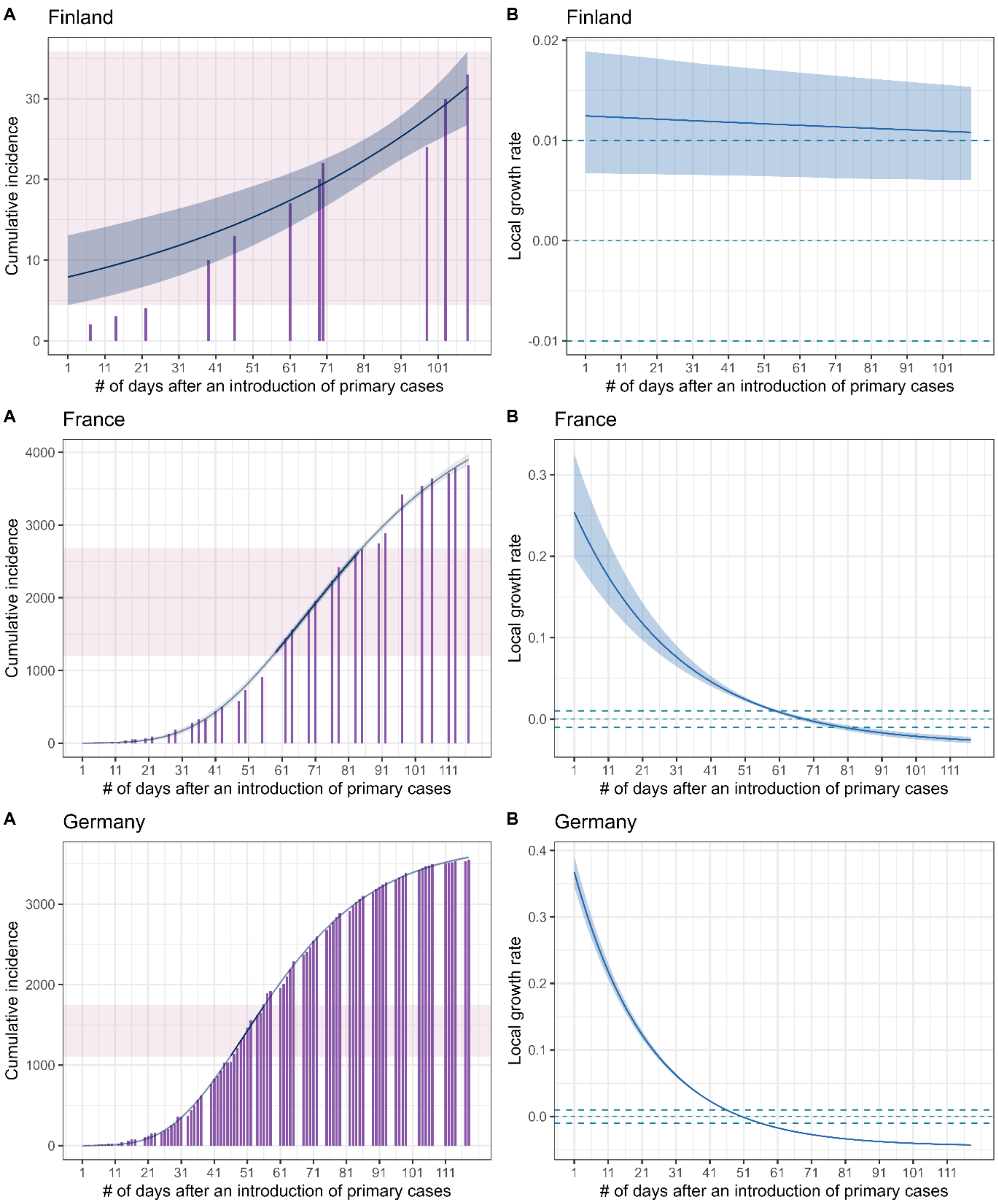
A
Finland
Cumulative incidence
0
10
20
30
1
11
21
31
41
51
61
71
81
91
101
# of days after an introduction of primary cases
B
Finland
Local growth rate
0.02
0.01
0.00
-0.01
1
11
21
31
41
51
61
71
81
91
101
# of days after an introduction of primary cases
A
France
Cumulative incidence
0
1000
2000
3000
4000
1
11
21
31
41
51
61
71
81
91
101
111
# of days after an introduction of primary cases
B
France
Local growth rate
0.0
0.1
0.2
0.3
1
11
21
31
41
51
61
71
81
91
101
111
# of days after an introduction of primary cases
A
Germany
Cumulative incidence
0
1000
2000
3000
1
11
21
31
41
51
61
71
81
91
101
111
# of days after an introduction of primary cases
B
Germany
Local growth rate
0.0
0.1
0.2
0.3
0.4
1
11
21
31
41
51
61
71
81
91
101
111
# of days after an introduction of primary cases

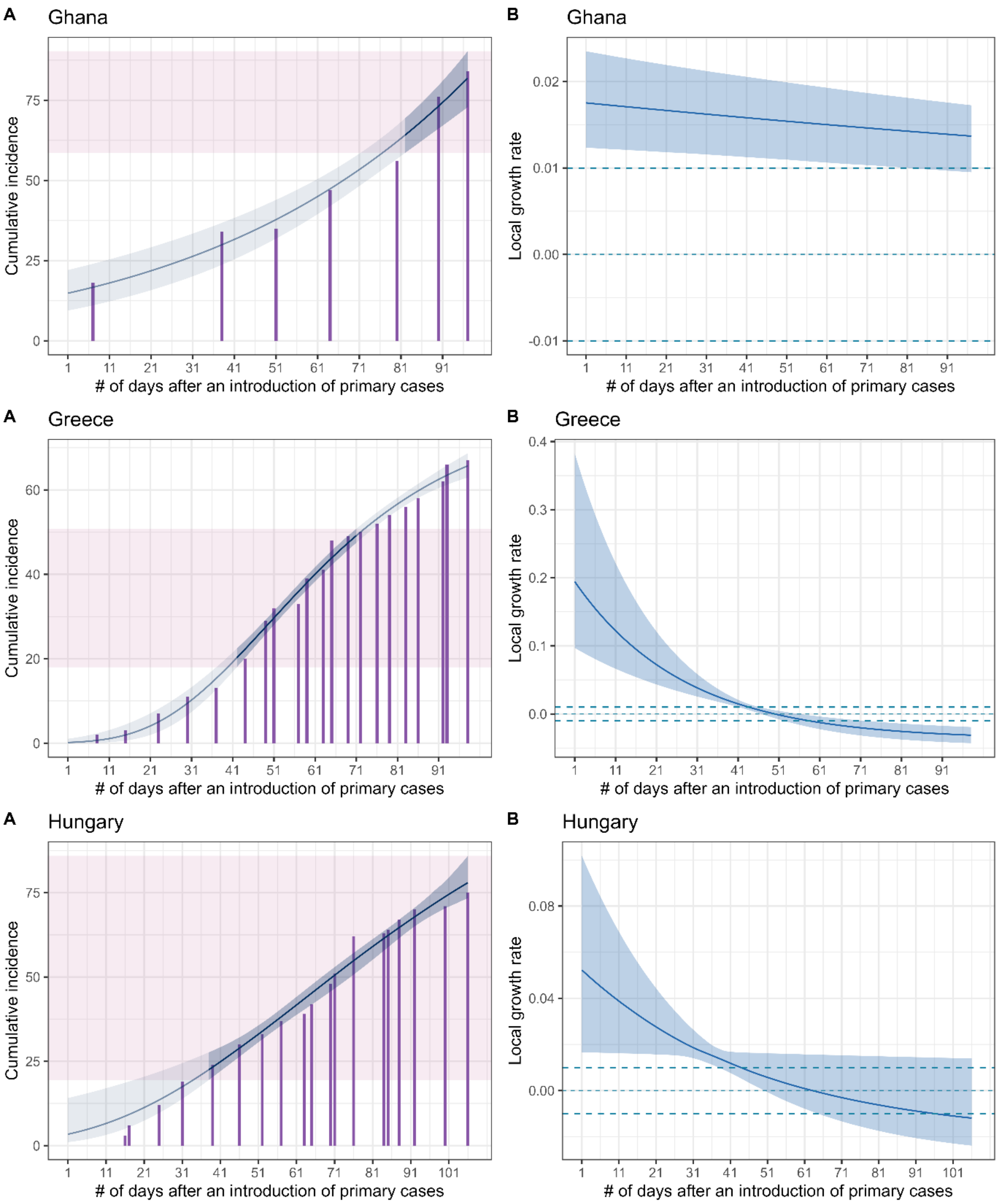
A
Ghana
Cumulative incidence
0
25
50
75
1
11
21
31
41
51
61
71
81
91
# of days after an introduction of primary cases
B
Ghana
Local growth rate
-0.01
0.00
0.01
0.02
1
11
21
31
41
51
61
71
81
91
# of days after an introduction of primary cases
A
Greece
Cumulative incidence
0
20
40
60
1
11
21
31
41
51
61
71
81
91
# of days after an introduction of primary cases
B
Greece
Local growth rate
0.0
0.1
0.2
0.3
0.4
1
11
21
31
41
51
61
71
81
91
# of days after an introduction of primary cases
A
Hungary
Cumulative incidence
0
25
50
75
1
11
21
31
41
51
61
71
81
91
101
# of days after an introduction of primary cases
B
Hungary
Local growth rate
0.00
0.04
0.08
1
11
21
31
41
51
61
71
81
91
101
# of days after an introduction of primary cases

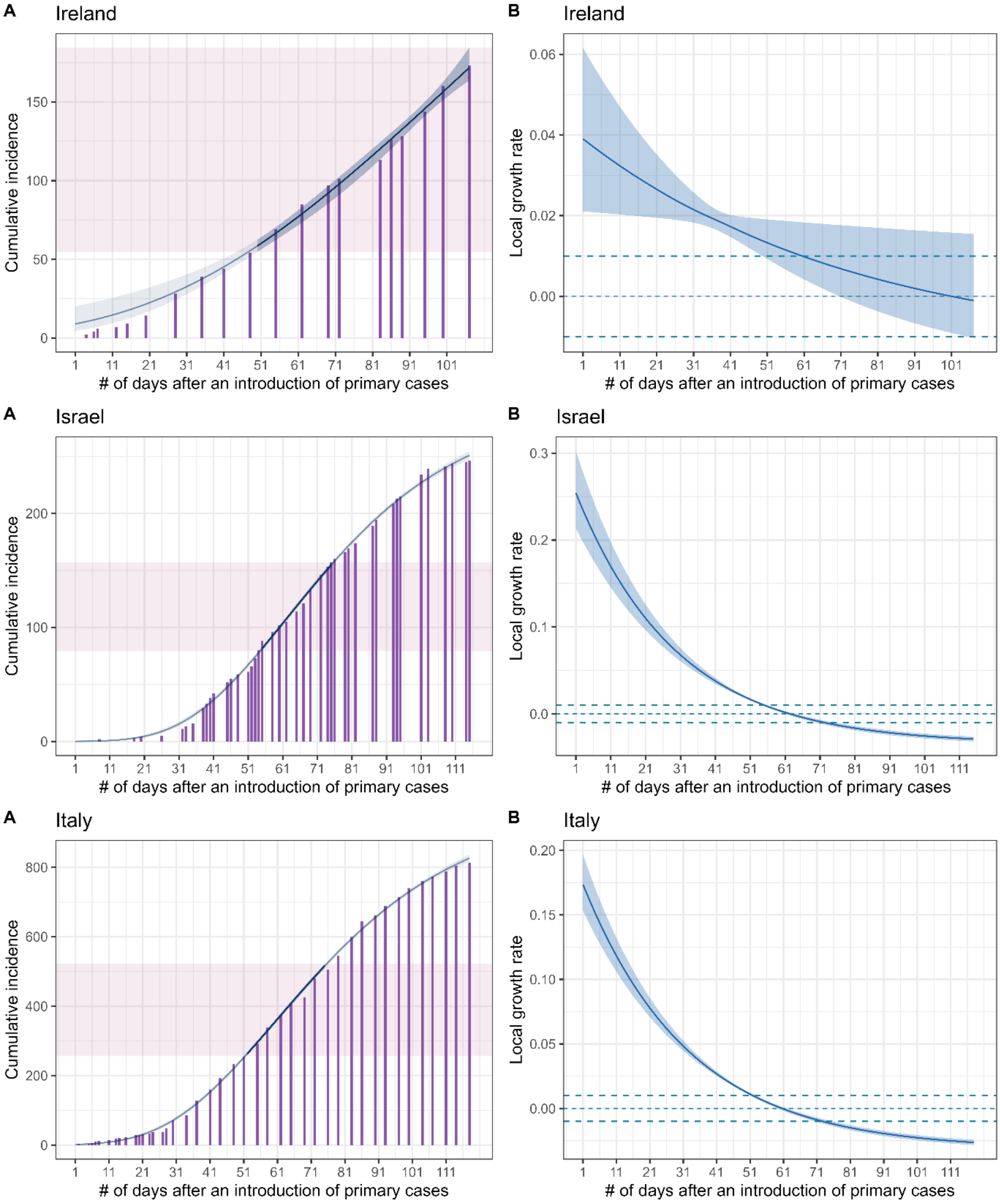

A
Ireland
Cumulative incidence
0
50
100
150
1
11
21
31
41
51
61
71
81
91
101
# of days after an introduction of primary cases
B
Ireland
Local growth rate
0.00
0.02
0.04
0.06
1
11
21
31
41
51
61
71
81
91
101
# of days after an introduction of primary cases
A
Israel
Cumulative incidence
0
100
200
1
11
21
31
41
51
61
71
81
91
101
111
# of days after an introduction of primary cases
B
Israel
Local growth rate
0.0
0.1
0.2
0.3
1
11
21
31
41
51
61
71
81
91
101
111
# of days after an introduction of primary cases
A
Italy
Cumulative incidence
0
200
400
600
800
1
11
21
31
41
51
61
71
81
91
101
111
# of days after an introduction of primary cases
B
Italy
Local growth rate
0.00
0.05
0.10
0.15
0.20
1
11
21
31
41
51
61
71
81
91
101
111
# of days after an introduction of primary cases

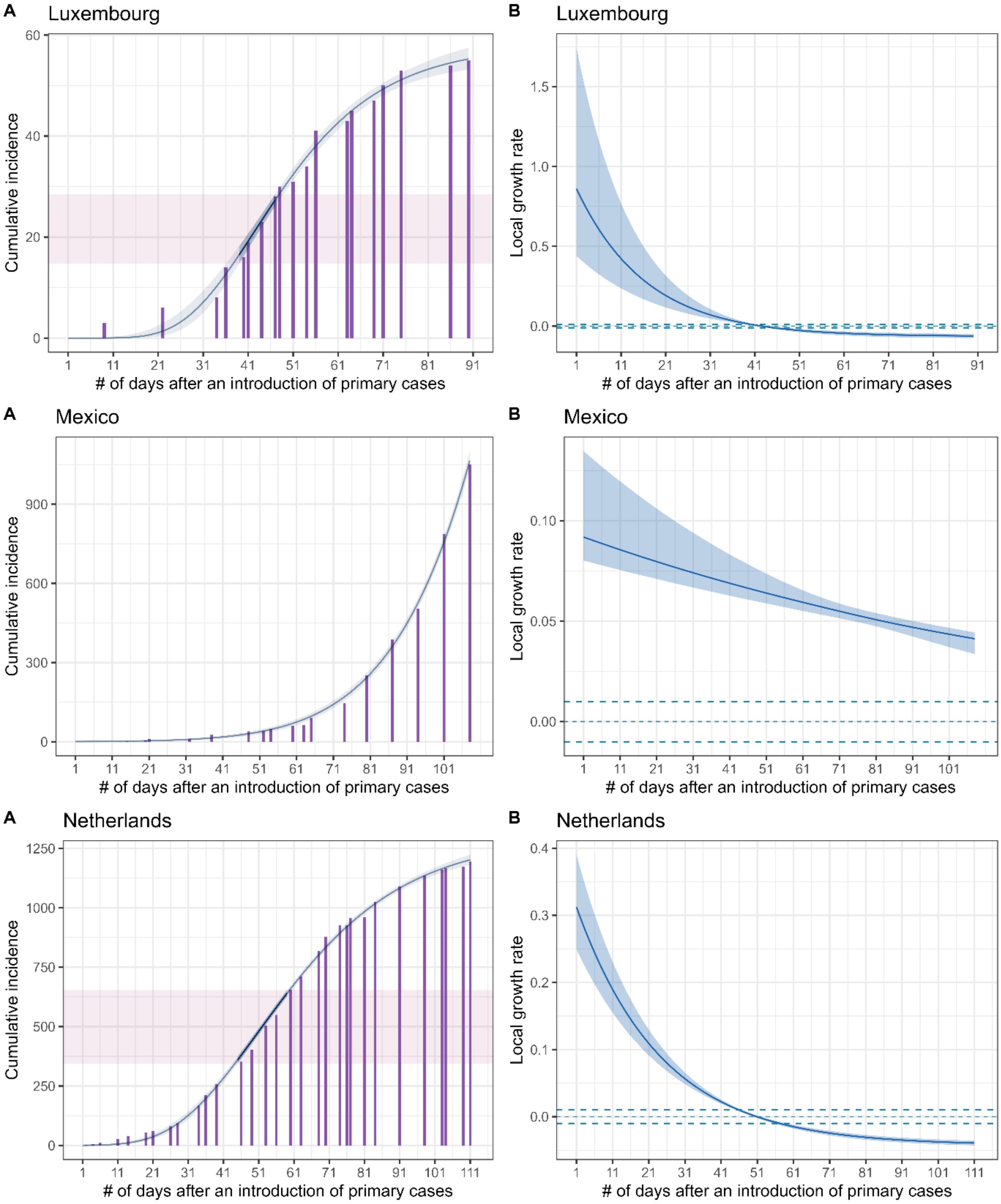
A
Luxembourg
Cumulative incidence
0
20
40
60
1
11
21
31
41
51
61
71
81
91
# of days after an introduction of primary cases
B
Luxembourg
Local growth rate
0.0
0.5
1.0
1.5
# of days after an introduction of primary cases
A
Mexico
Cumulative incidence
0
300
600
900
101
# of days after an introduction of primary cases
B
Mexico
Local growth rate
0.00
0.05
0.10
# of days after an introduction of primary cases
A
Netherlands
Cumulative incidence
0
250
500
750
1000
1250
111
# of days after an introduction of primary cases
B
Netherlands
Local growth rate
0.0
0.1
0.2
0.3
0.4
# of days after an introduction of primary cases

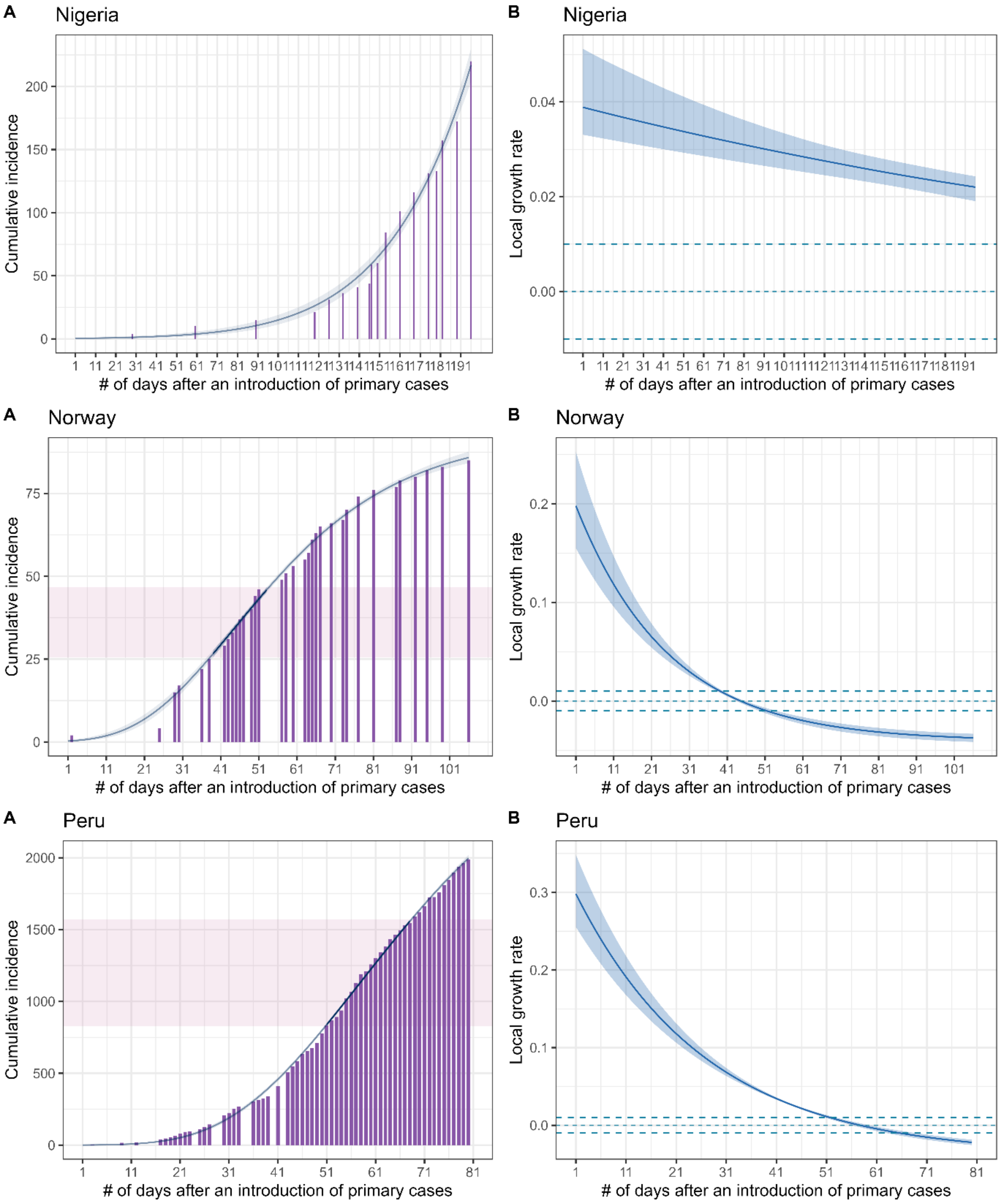

A
Nigeria
Cumulative incidence
0
50
100
150
200
# of days after an introduction of primary cases
B
Nigeria
Local growth rate
0.00
0.02
0.04
# of days after an introduction of primary cases
A
Norway
Cumulative incidence
0
25
50
75
1
11
21
31
41
51
61
71
81
91
101
# of days after an introduction of primary cases
B
Norway
Local growth rate
0.0
0.1
0.2
1
11
21
31
41
51
61
71
81
91
101
# of days after an introduction of primary cases
A
Peru
Cumulative incidence
0
500
1000
1500
2000
1
11
21
31
41
51
61
71
81
# of days after an introduction of primary cases
B
Peru
Local growth rate
0.0
0.1
0.2
0.3
1
11
21
31
41
51
61
71
81
# of days after an introduction of primary cases

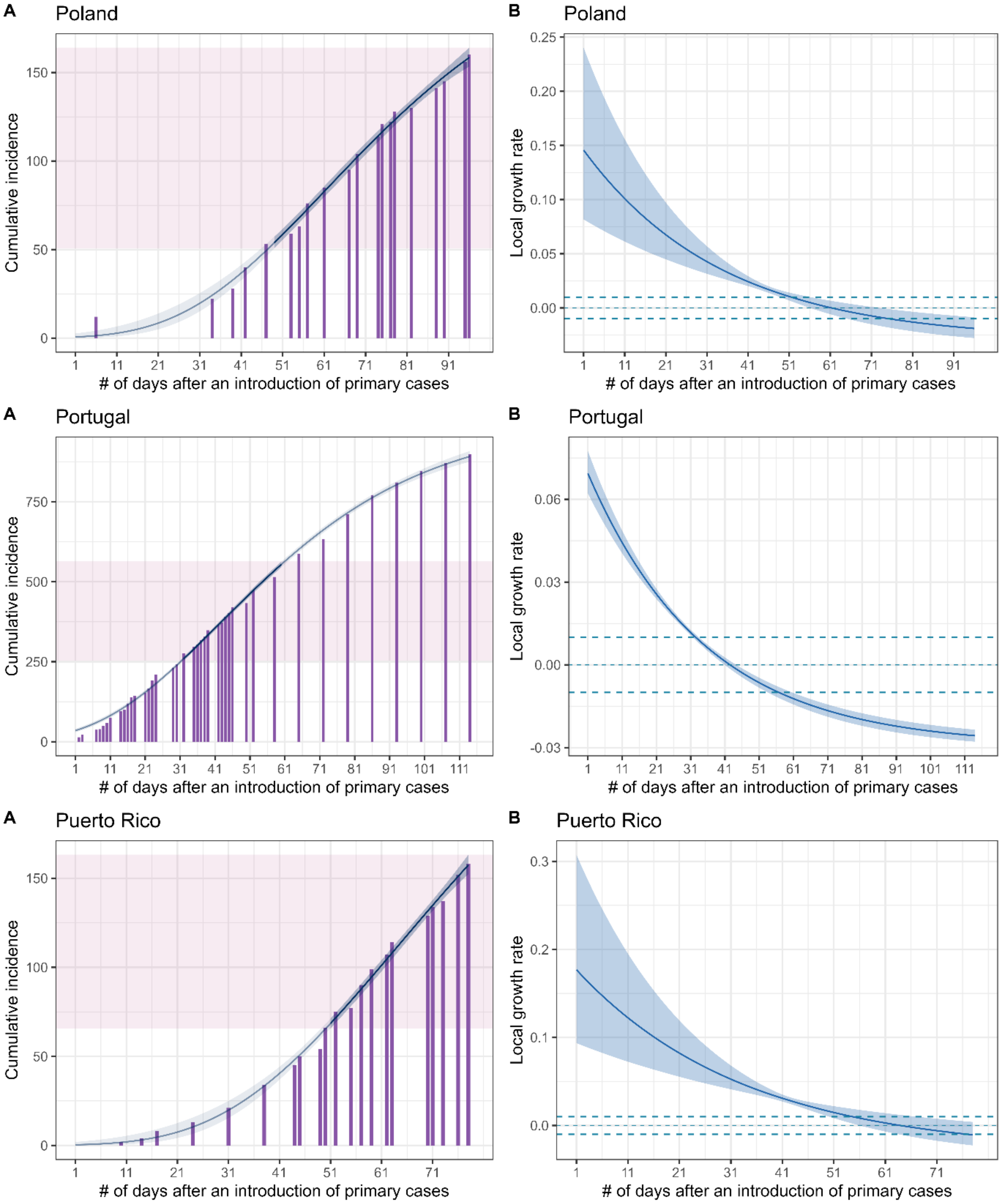
A
Poland
Cumulative incidence
0
50
100
150
1
11
21
31
41
51
61
71
81
91
# of days after an introduction of primary cases
B
Poland
Local growth rate
0.00
0.05
0.10
0.15
0.20
0.25
1
11
21
31
41
51
61
71
81
91
# of days after an introduction of primary cases
A
Portugal
Cumulative incidence
0
250
500
750
1
11
21
31
41
51
61
71
81
91
101
111
# of days after an introduction of primary cases
B
Portugal
Local growth rate
-0.03
0.00
0.03
0.06
1
11
21
31
41
51
61
71
81
91
101
111
# of days after an introduction of primary cases
A
Puerto Rico
Cumulative incidence
0
50
100
150
1
11
21
31
41
51
61
71
# of days after an introduction of primary cases
B
Puerto Rico
Local growth rate
0.0
0.1
0.2
0.3
1
11
21
31
41
51
61
71
# of days after an introduction of primary cases

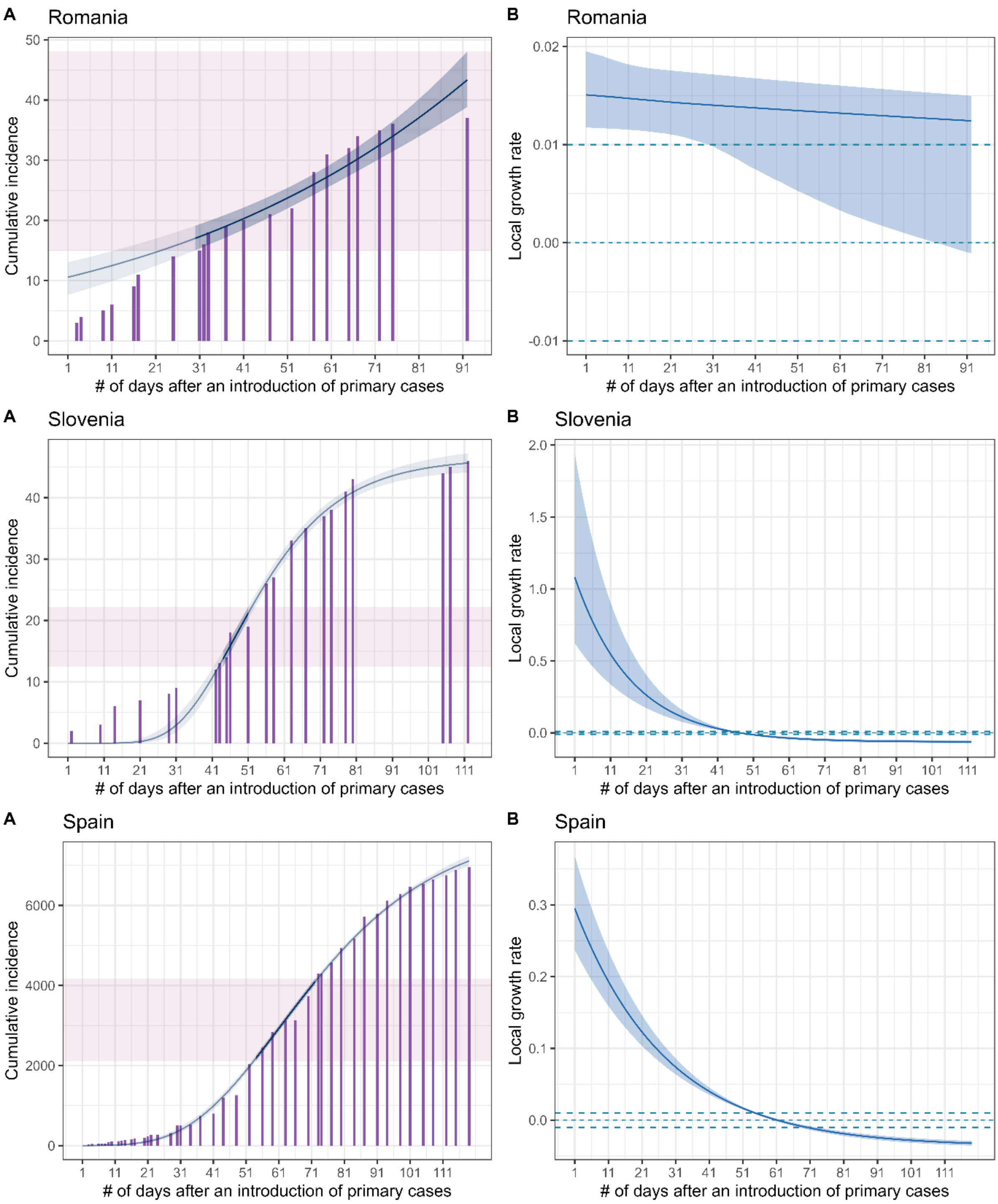
A
Romania
Cumulative incidence
0
10
20
30
40
50
1 11 21 31 41 51 61 71 81 91
# of days after an introduction of primary cases
B
Romania
Local growth rate
-0.01
0.00
0.01
0.02
1 11 21 31 41 51 61 71 81 91
# of days after an introduction of primary cases
A
Slovenia
Cumulative incidence
0
10
20
30
40
1 11 21 31 41 51 61 71 81 91 101 111
# of days after an introduction of primary cases
B
Slovenia
Local growth rate
0.0
0.5
1.0
1.5
2.0
1 11 21 31 41 51 61 71 81 91 101 111
# of days after an introduction of primary cases
A
Spain
Cumulative incidence
0
2000
4000
6000
1 11 21 31 41 51 61 71 81 91 101 111
# of days after an introduction of primary cases
B
Spain
Local growth rate
0.0
0.1
0.2
0.3
1 11 21 31 41 51 61 71 81 91 101 111
# of days after an introduction of primary cases

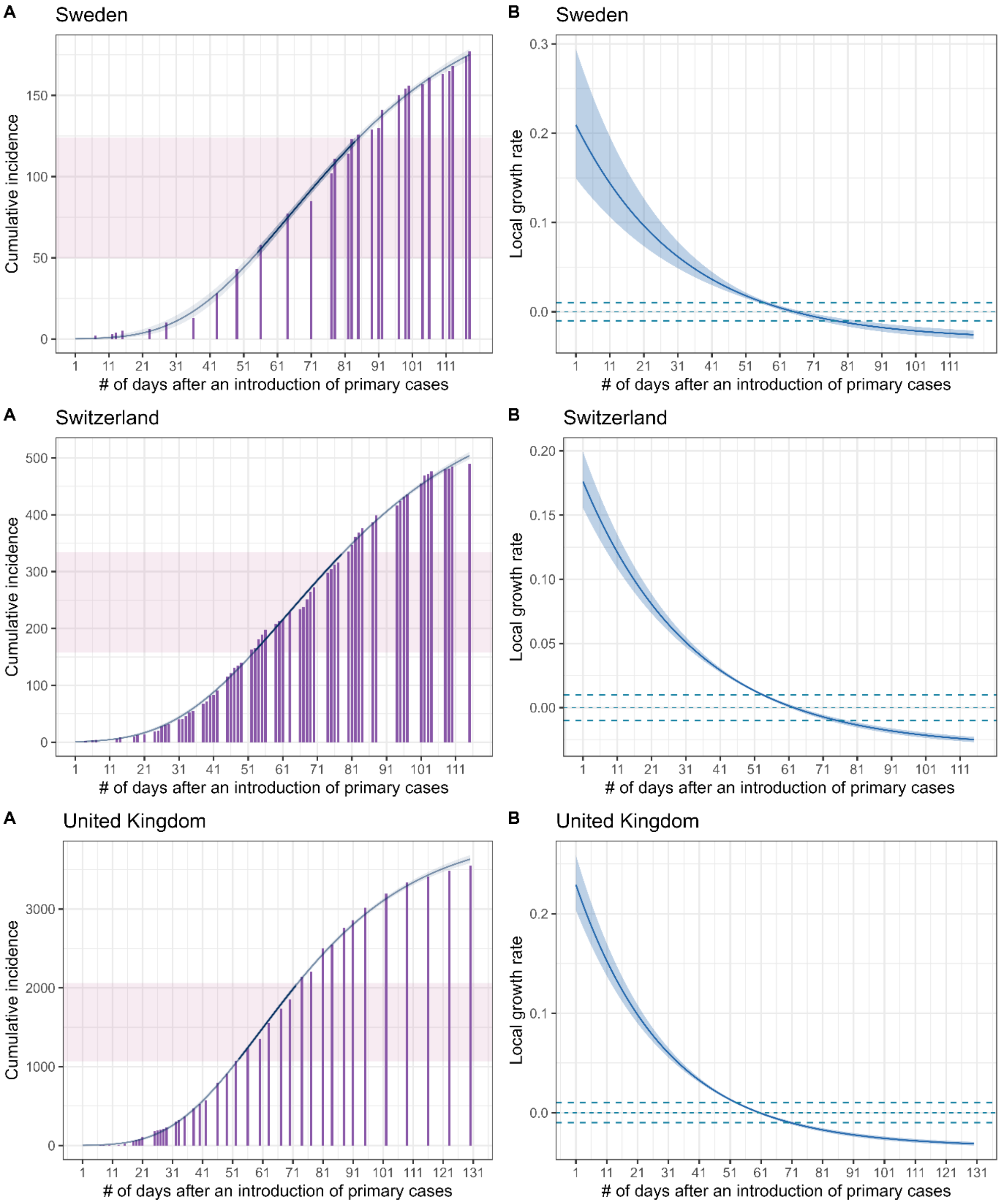

A
Sweden
Cumulative incidence
0
50
100
150
1
11
21
31
41
51
61
71
81
91
101
111
# of days after an introduction of primary cases
B
Sweden
Local growth rate
0.0
0.1
0.2
0.3
1
11
21
31
41
51
61
71
81
91
101
111
# of days after an introduction of primary cases
A
Switzerland
Cumulative incidence
0
100
200
300
400
500
1
11
21
31
41
51
61
71
81
91
101
111
# of days after an introduction of primary cases
B
Switzerland
Local growth rate
0.00
0.05
0.10
0.15
0.20
1
11
21
31
41
51
61
71
81
91
101
111
# of days after an introduction of primary cases
A
United Kingdom
Cumulative incidence
0
1000
2000
3000
1
11
21
31
41
51
61
71
81
91
101
111
121
131
# of days after an introduction of primary cases
B
United Kingdom
Local growth rate
0.0
0.1
0.2
1
11
21
31
41
51
61
71
81
91
101
111
121
131
# of days after an introduction of primary cases

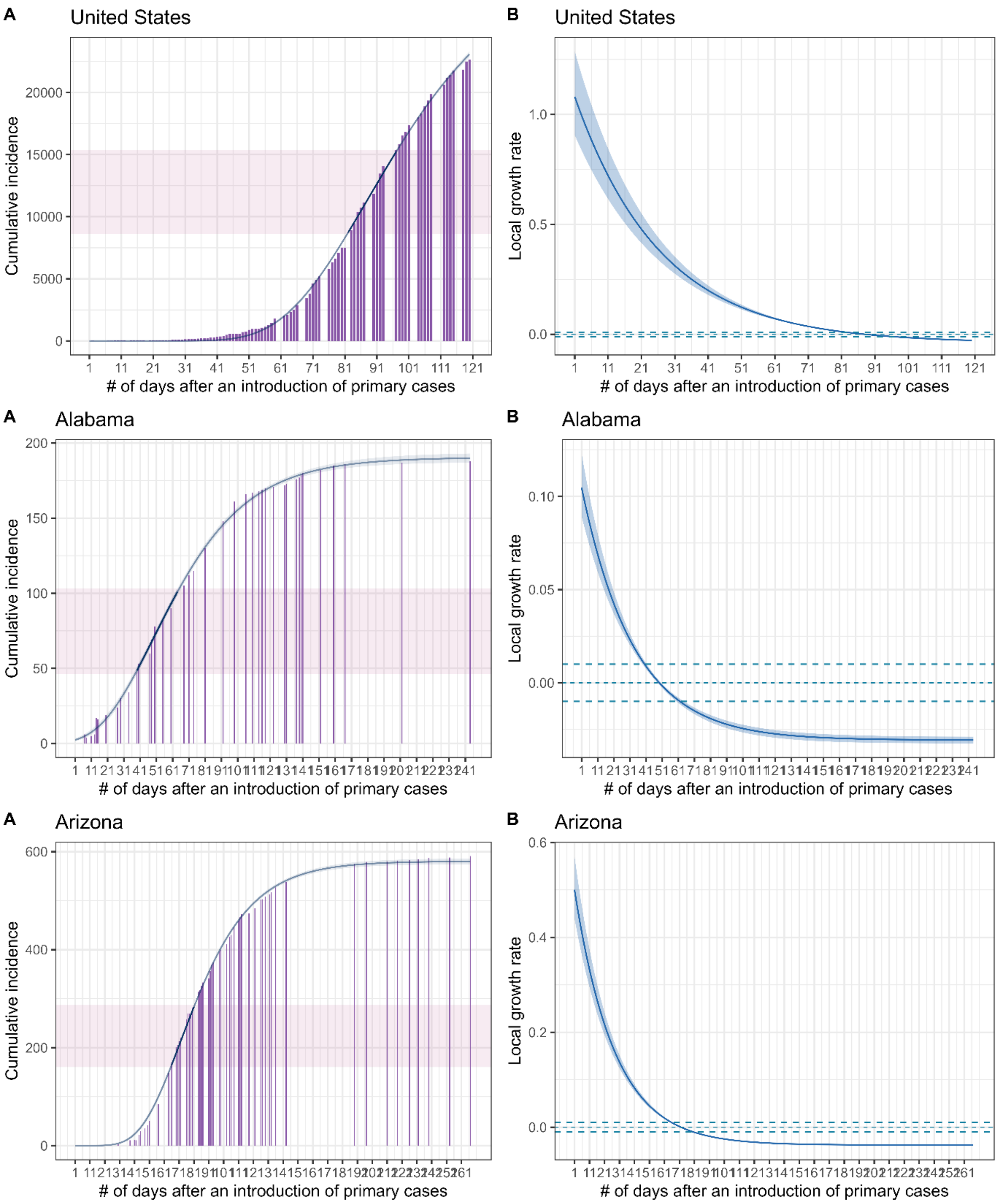
A
United States
Cumulative incidence
20000
15000
10000
5000
0
1
11
21
31
41
51
61
71
81
91
101
111
121
# of days after an introduction of primary cases
B
United States
Local growth rate
1.0
0.5
0.0
1
11
21
31
41
51
61
71
81
91
101
111
121
# of days after an introduction of primary cases
A
Alabama
Cumulative incidence
200
150
100
50
0
# of days after an introduction of primary cases
B
Alabama
Local growth rate
0.10
0.05
0.00
# of days after an introduction of primary cases
A
Arizona
Cumulative incidence
600
400
200
0
# of days after an introduction of primary cases
B
Arizona
Local growth rate
0.6
0.4
0.2
0.0
# of days after an introduction of primary cases

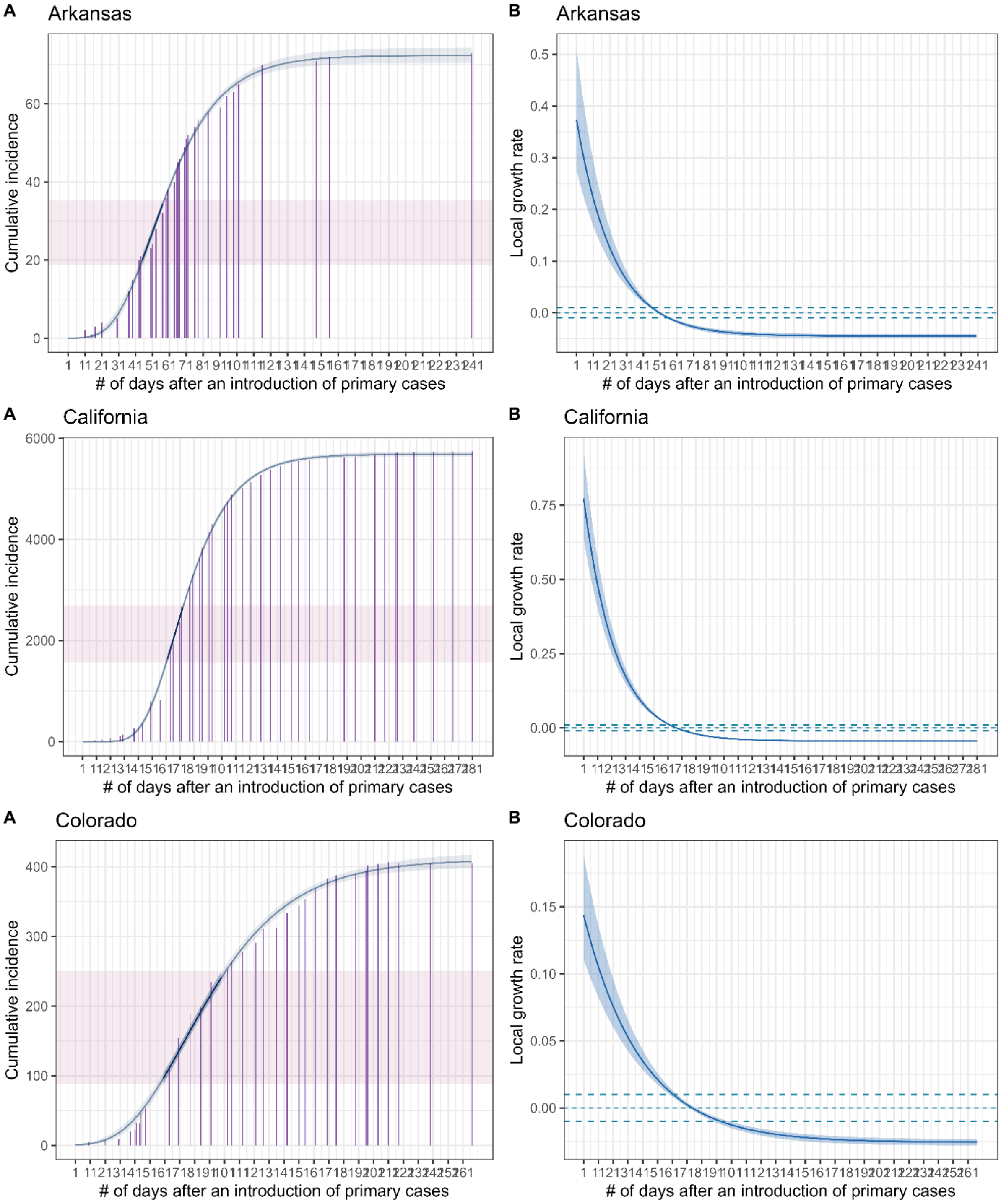
A
Arkansas
Cumulative incidence
0
20
40
60
# of days after an introduction of primary cases
B
Arkansas
Local growth rate
0.0
0.1
0.2
0.3
0.4
0.5
# of days after an introduction of primary cases
A
California
Cumulative incidence
0
2000
4000
6000
# of days after an introduction of primary cases
B
California
Local growth rate
0.00
0.25
0.50
0.75
# of days after an introduction of primary cases
A
Colorado
Cumulative incidence
0
100
200
300
400
# of days after an introduction of primary cases
B
Colorado
Local growth rate
0.00
0.05
0.10
0.15
# of days after an introduction of primary cases

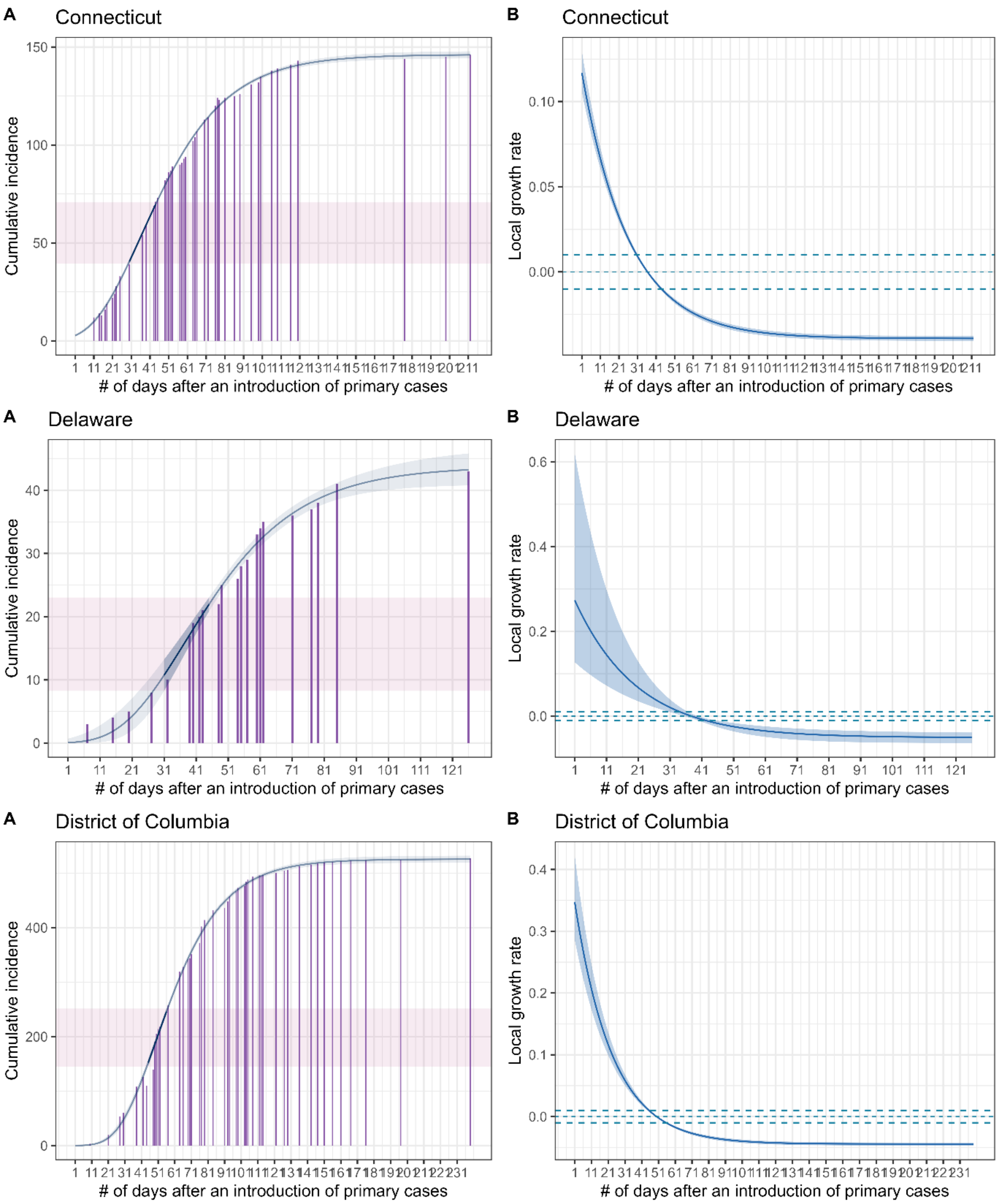

A
Connecticut
Cumulative incidence
0
50
100
150
# of days after an introduction of primary cases
B
Connecticut
Local growth rate
0.00
0.05
0.10
# of days after an introduction of primary cases
A
Delaware
Cumulative incidence
0
10
20
30
40
1 11 21 31 41 51 61 71 81 91 101 111 121
# of days after an introduction of primary cases
B
Delaware
Local growth rate
0.0
0.2
0.4
0.6
1 11 21 31 41 51 61 71 81 91 101 111 121
# of days after an introduction of primary cases
A
District of Columbia
Cumulative incidence
0
200
400
# of days after an introduction of primary cases
B
District of Columbia
Local growth rate
0.0
0.1
0.2
0.3
0.4
# of days after an introduction of primary cases

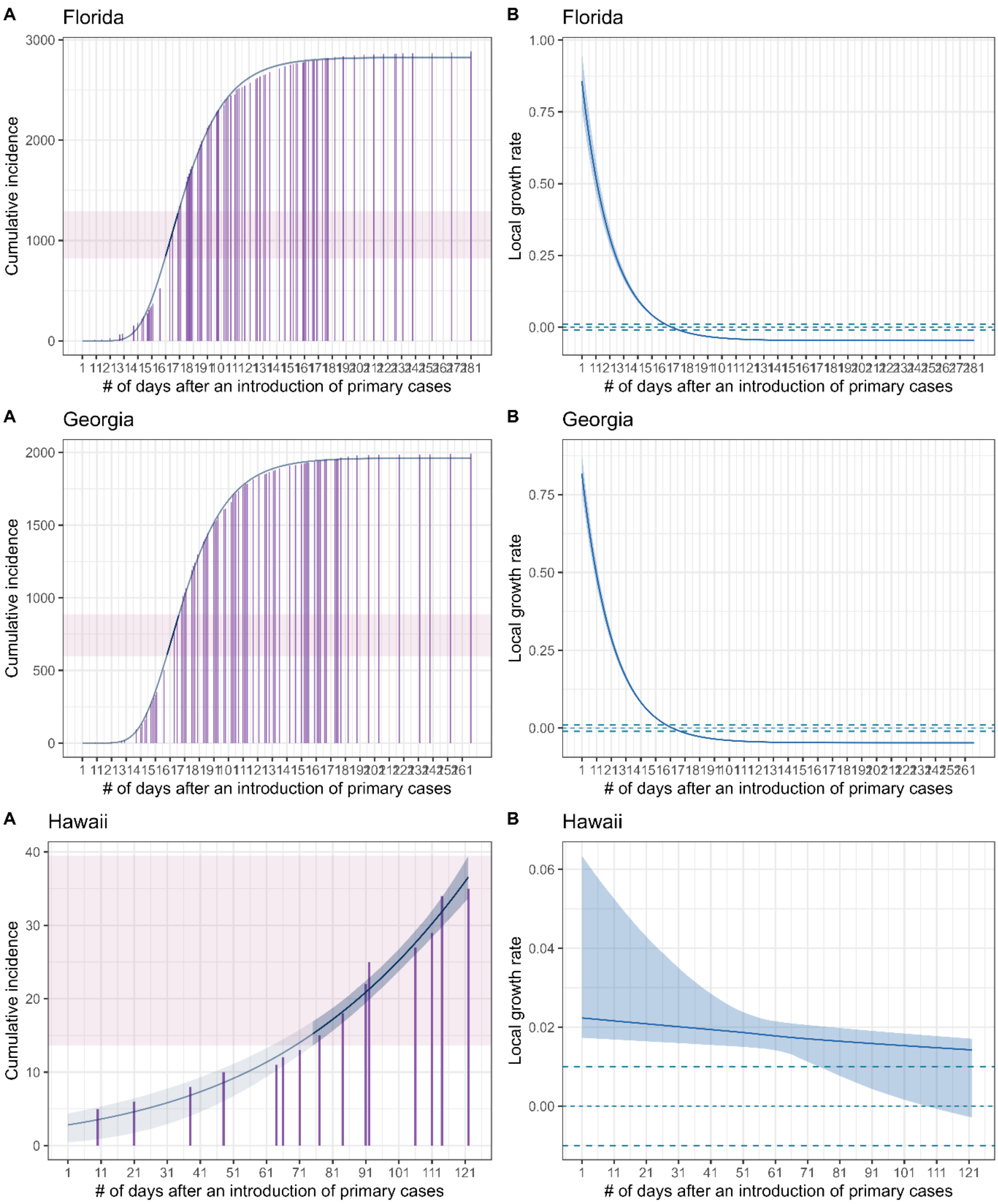
A
Florida
Cumulative incidence
3000
2000
1000
0
# of days after an introduction of primary cases
B
Florida
Local growth rate
1.00
0.75
0.50
0.25
0.00
# of days after an introduction of primary cases
A
Georgia
Cumulative incidence
2000
1500
1000
500
0
# of days after an introduction of primary cases
B
Georgia
Local growth rate
0.75
0.50
0.25
0.00
# of days after an introduction of primary cases
A
Hawaii
Cumulative incidence
40
30
20
10
0
1 11 21 31 41 51 61 71 81 91 101 111 121
# of days after an introduction of primary cases
B
Hawaii
Local growth rate
0.06
0.04
0.02
0.00
1 11 21 31 41 51 61 71 81 91 101 111 121
# of days after an introduction of primary cases

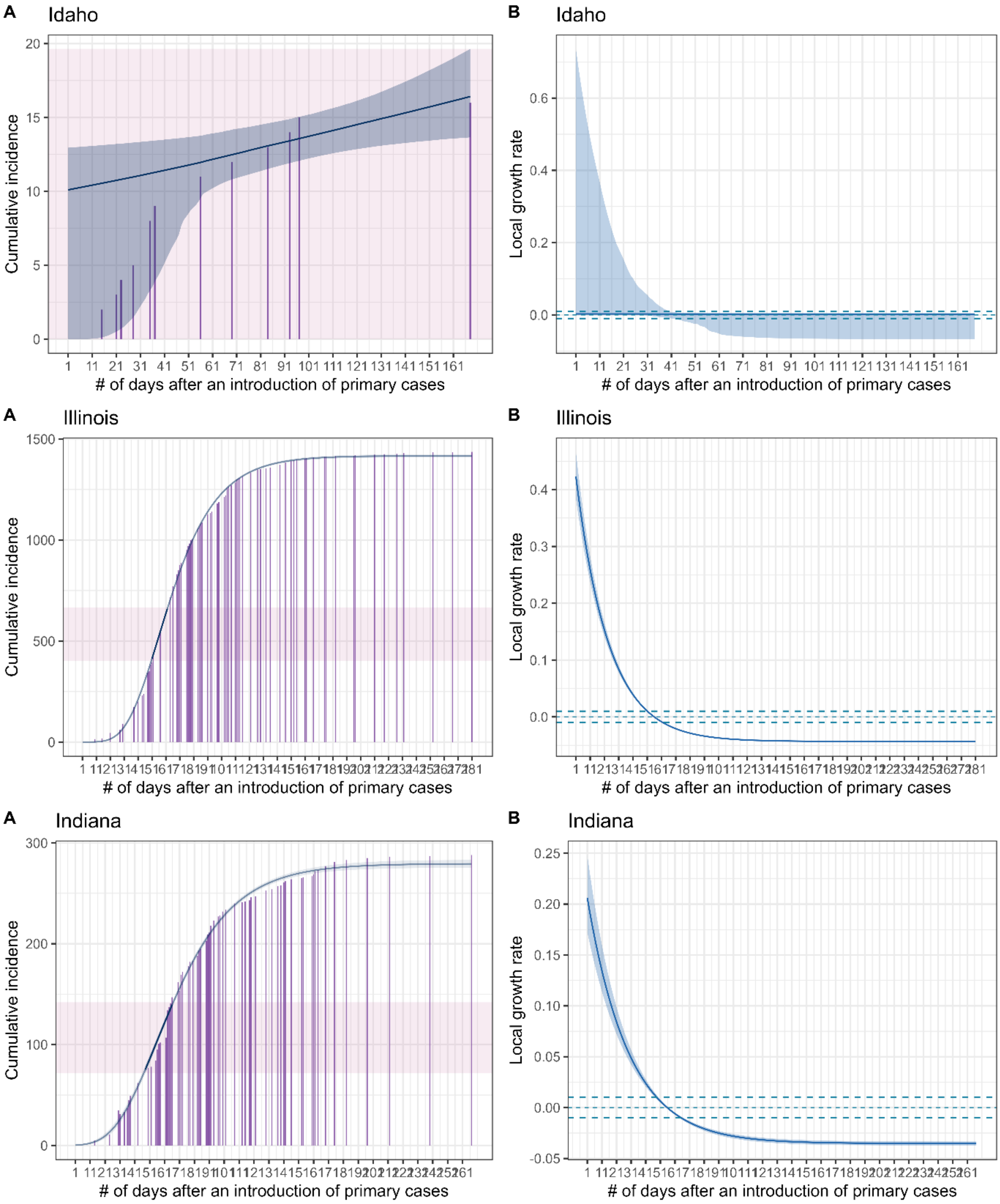
A
Idaho
Cumulative incidence
# of days after an introduction of primary cases
B
Idaho
Local growth rate
# of days after an introduction of primary cases
A
Illinois
Cumulative incidence
# of days after an introduction of primary cases
B
Illinois
Local growth rate
# of days after an introduction of primary cases
A
Indiana
Cumulative incidence
# of days after an introduction of primary cases
B
Indiana
Local growth rate
# of days after an introduction of primary cases

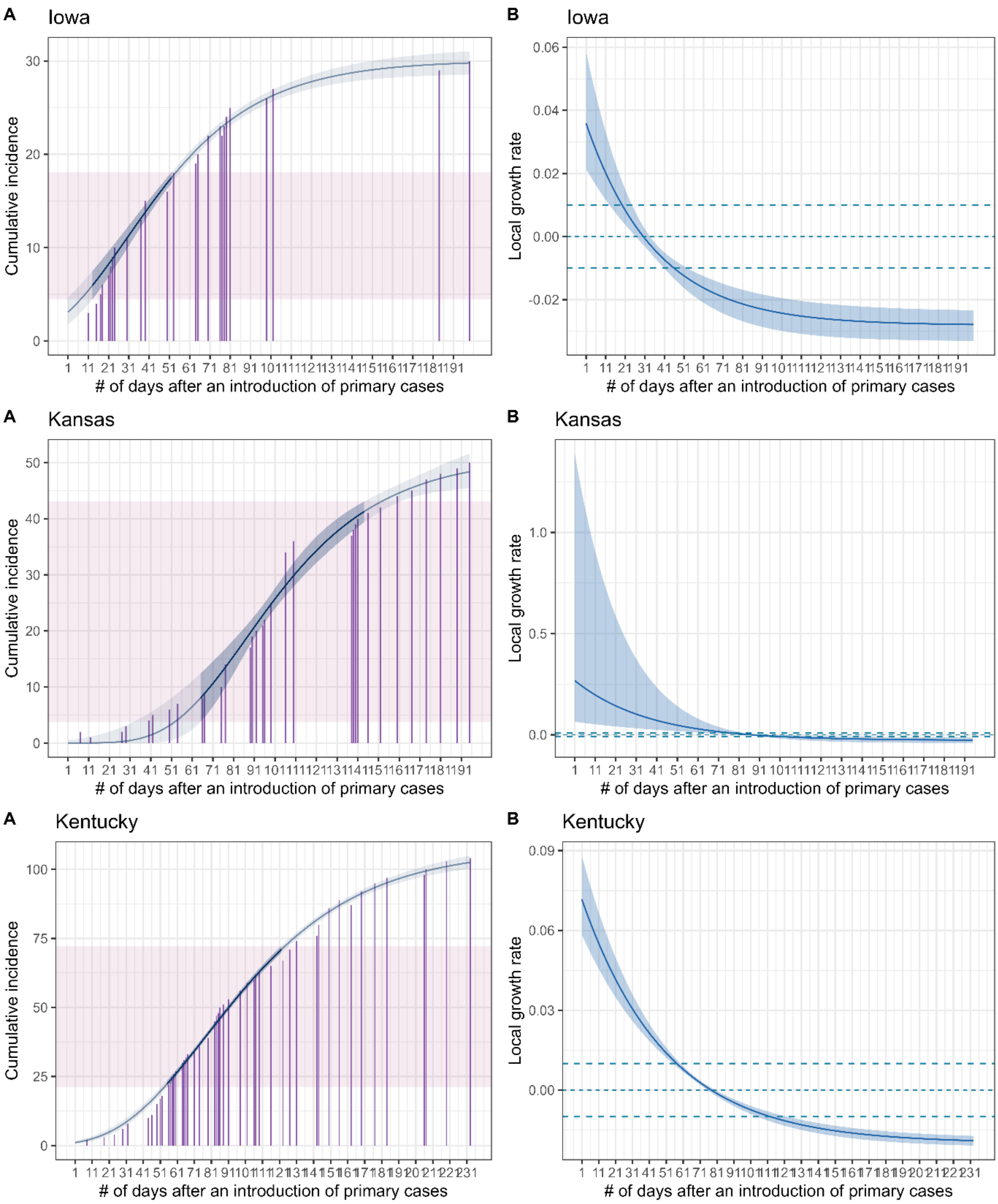
A
Iowa
Cumulative incidence
# of days after an introduction of primary cases
B
Iowa
Local growth rate
# of days after an introduction of primary cases
A
Kansas
Cumulative incidence
# of days after an introduction of primary cases
B
Kansas
Local growth rate
# of days after an introduction of primary cases
A
Kentucky
Cumulative incidence
# of days after an introduction of primary cases
B
Kentucky
Local growth rate
# of days after an introduction of primary cases

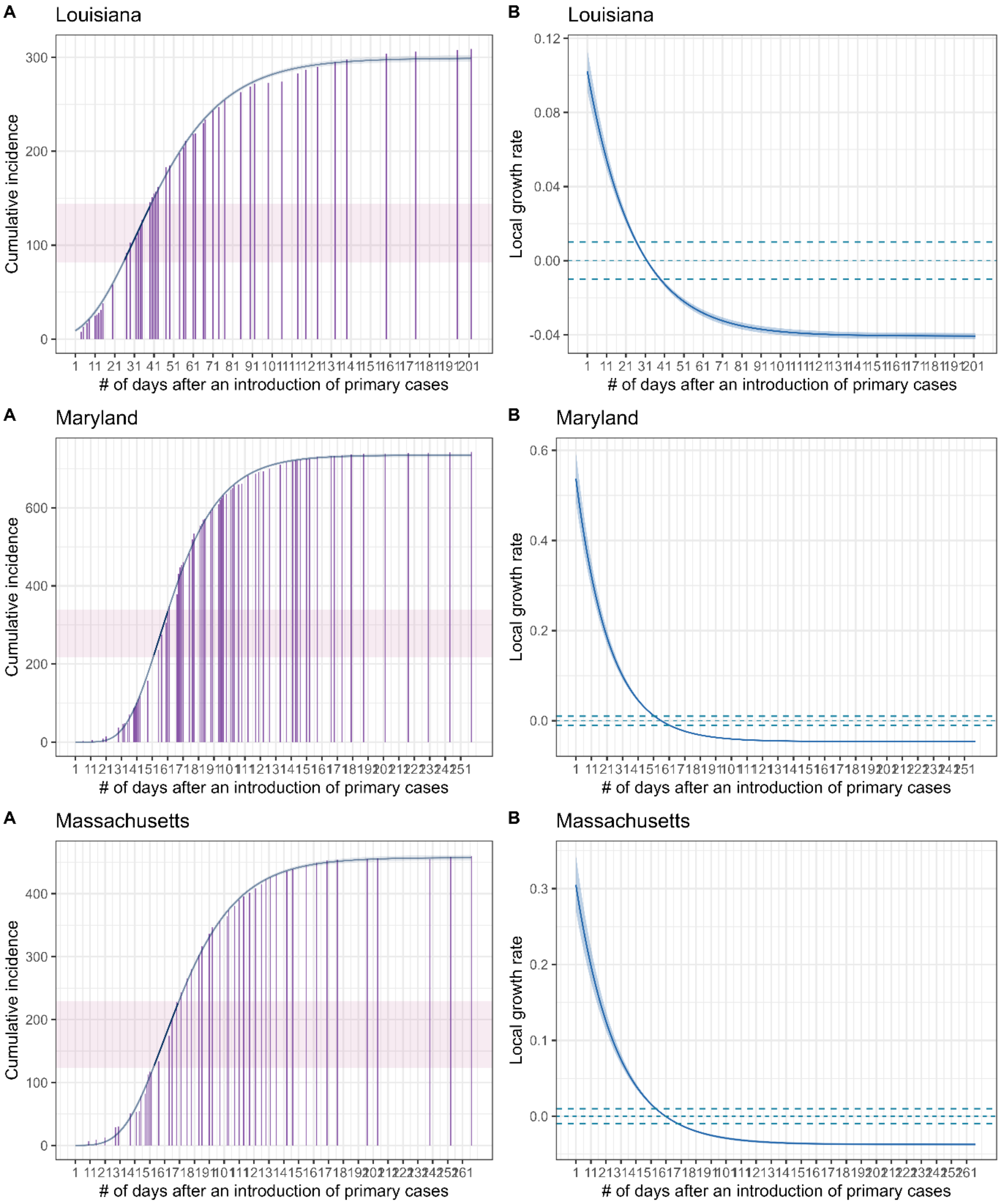
A
Louisiana
Cumulative incidence
0
100
200
300
# of days after an introduction of primary cases
B
Louisiana
Local growth rate
0.12
0.08
0.04
0.00
-0.04
# of days after an introduction of primary cases
A
Maryland
Cumulative incidence
0
200
400
600
# of days after an introduction of primary cases
B
Maryland
Local growth rate
0.6
0.4
0.2
0.0
# of days after an introduction of primary cases
A
Massachusetts
Cumulative incidence
0
100
200
300
400
# of days after an introduction of primary cases
B
Massachusetts
Local growth rate
0.3
0.2
0.1
0.0
# of days after an introduction of primary cases

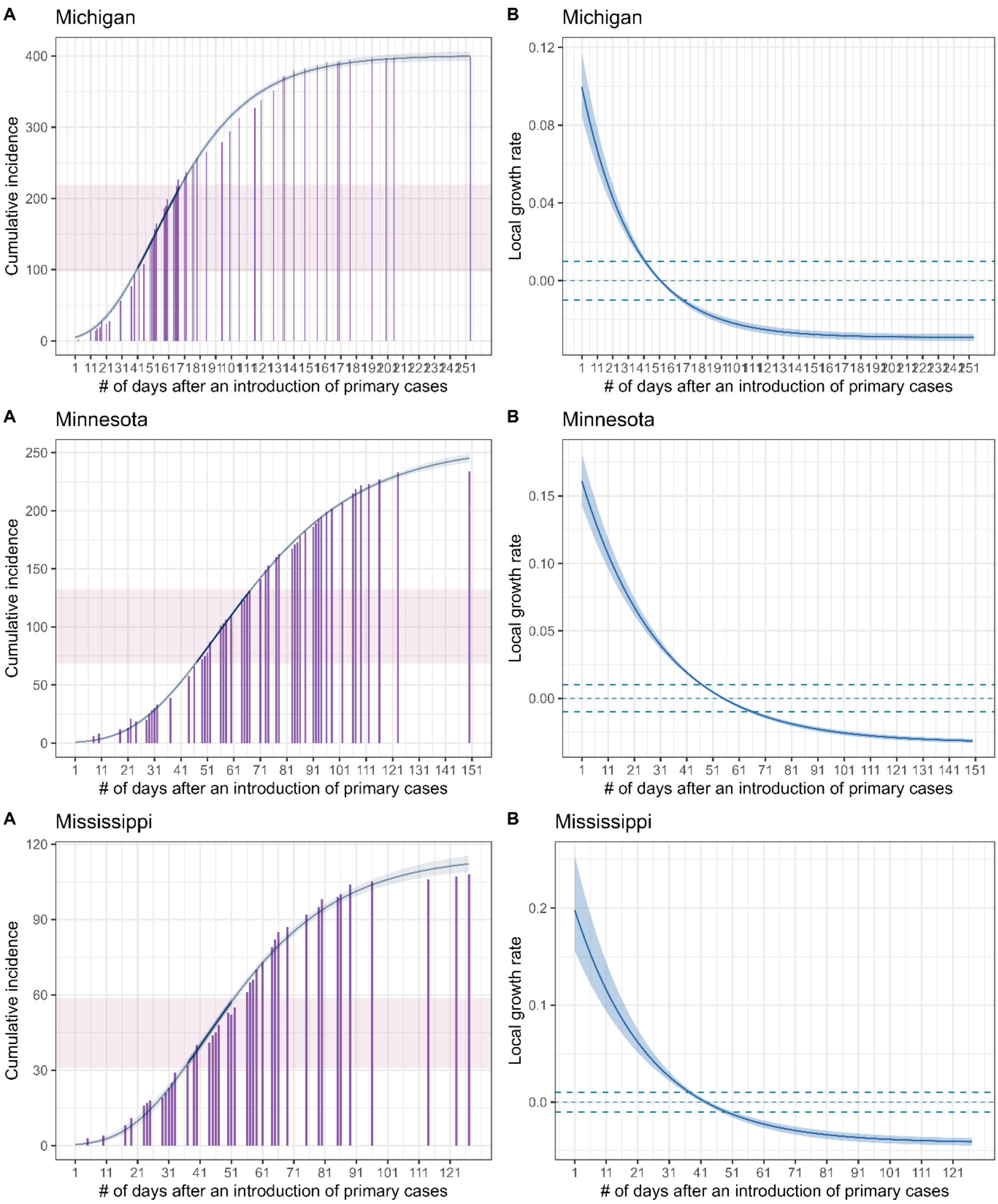
A
Michigan
Cumulative incidence
0
100
200
300
400
# of days after an introduction of primary cases
B
Michigan
Local growth rate
0.00
0.04
0.08
0.12
# of days after an introduction of primary cases
A
Minnesota
Cumulative incidence
0
50
100
150
200
250
1 11 21 31 41 51 61 71 81 91 101 111 121 131 141 151
# of days after an introduction of primary cases
B
Minnesota
Local growth rate
0.00
0.05
0.10
0.15
1 11 21 31 41 51 61 71 81 91 101 111 121 131 141 151
# of days after an introduction of primary cases
A
Mississippi
Cumulative incidence
0
30
60
90
120
1 11 21 31 41 51 61 71 81 91 101 111 121
# of days after an introduction of primary cases
B
Mississippi
Local growth rate
0.0
0.1
0.2
1 11 21 31 41 51 61 71 81 91 101 111 121
# of days after an introduction of primary cases

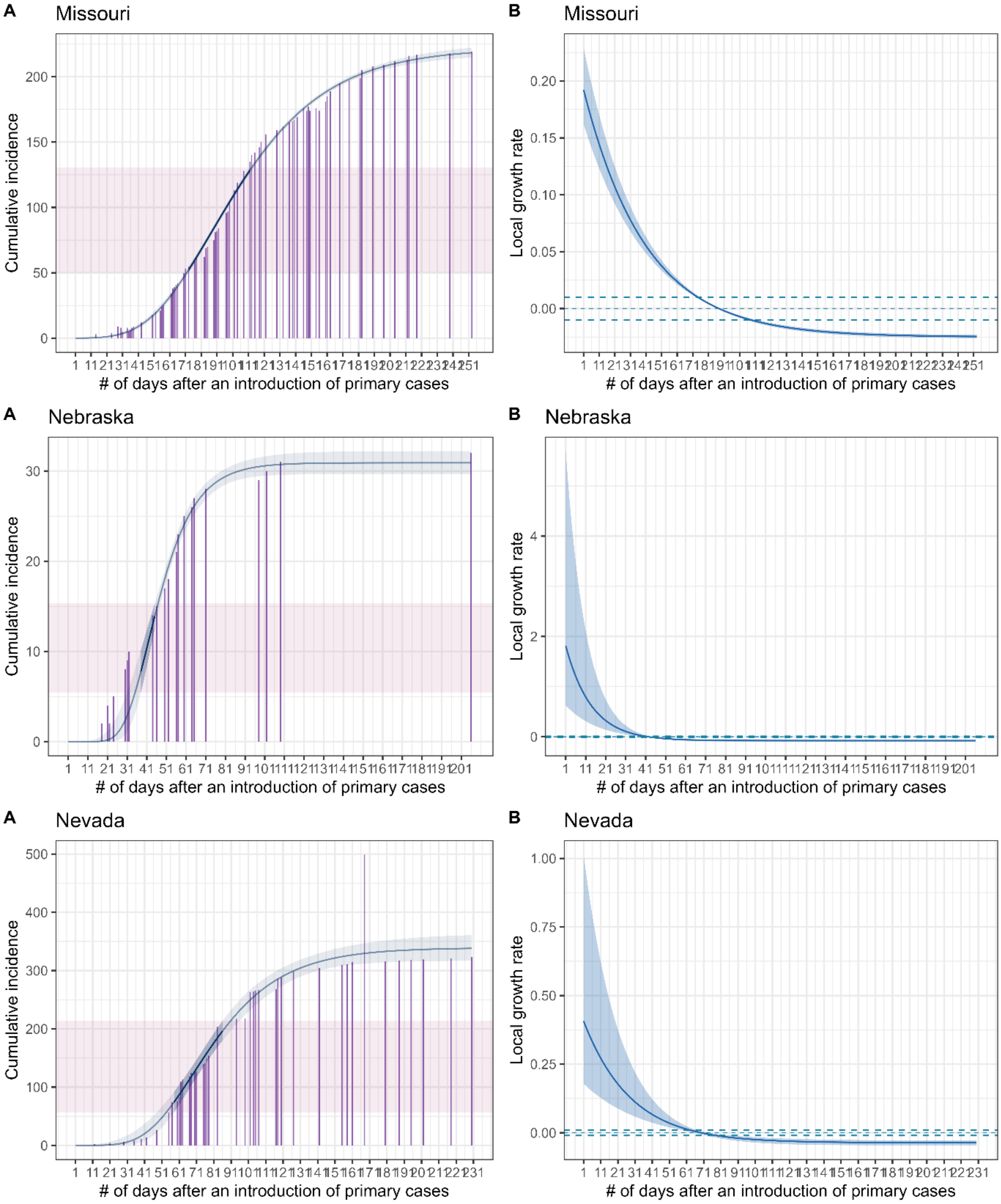
A
Missouri
Cumulative incidence
0
50
100
150
200
# of days after an introduction of primary cases
B
Missouri
Local growth rate
0.00
0.05
0.10
0.15
0.20
# of days after an introduction of primary cases
A
Nebraska
Cumulative incidence
0
10
20
30
# of days after an introduction of primary cases
B
Nebraska
Local growth rate
0
2
4
# of days after an introduction of primary cases
A
Nevada
Cumulative incidence
0
100
200
300
400
500
# of days after an introduction of primary cases
B
Nevada
Local growth rate
0.00
0.25
0.50
0.75
1.00
# of days after an introduction of primary cases

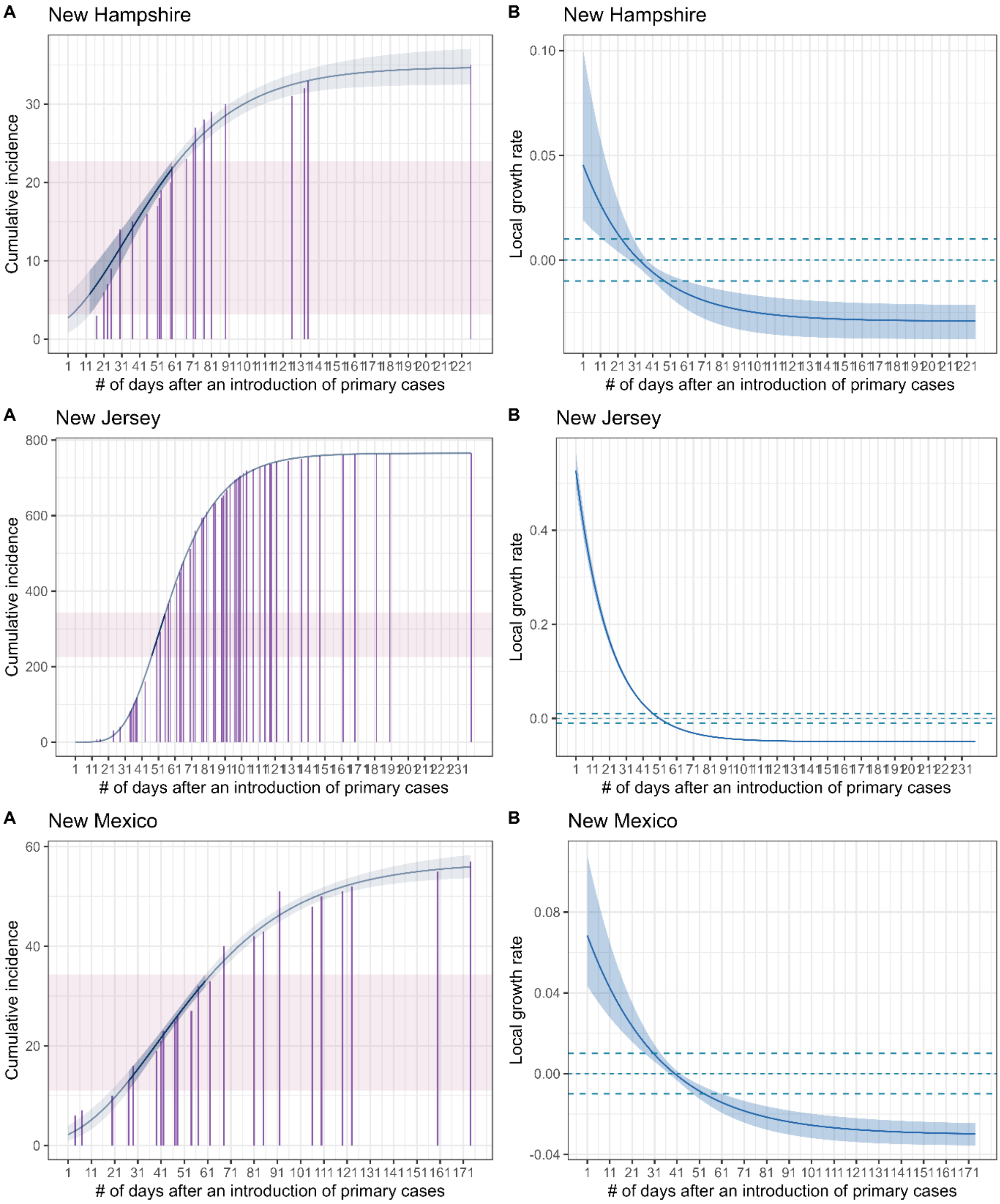

A
New Hampshire
Cumulative incidence
0
10
20
30
# of days after an introduction of primary cases
B
New Hampshire
Local growth rate
0.10
0.05
0.00
# of days after an introduction of primary cases
A
New Jersey
Cumulative incidence
0
200
400
600
800
# of days after an introduction of primary cases
B
New Jersey
Local growth rate
0.4
0.2
0.0
# of days after an introduction of primary cases
A
New Mexico
Cumulative incidence
0
20
40
60
1 11 21 31 41 51 61 71 81 91 101 111 121 131 141 151 161 171
# of days after an introduction of primary cases
B
New Mexico
Local growth rate
0.08
0.04
0.00
-0.04
1 11 21 31 41 51 61 71 81 91 101 111 121 131 141 151 161 171
# of days after an introduction of primary cases

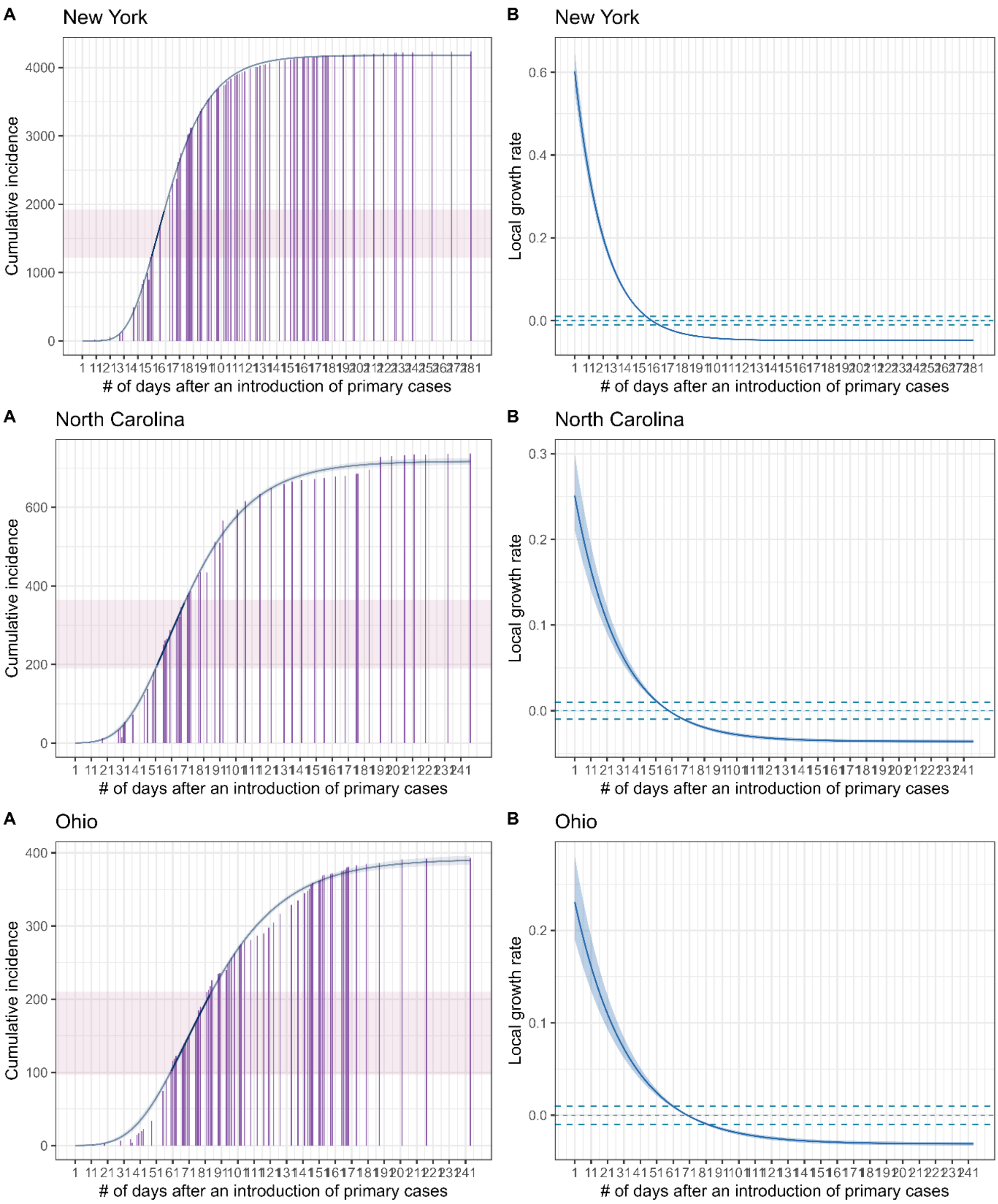
A
New York
Cumulative incidence
4000
3000
2000
1000
0
# of days after an introduction of primary cases
B
New York
Local growth rate
0.6
0.4
0.2
0.0
# of days after an introduction of primary cases
A
North Carolina
Cumulative incidence
600
400
200
0
# of days after an introduction of primary cases
B
North Carolina
Local growth rate
0.3
0.2
0.1
0.0
# of days after an introduction of primary cases
A
Ohio
Cumulative incidence
400
300
200
100
0
# of days after an introduction of primary cases
B
Ohio
Local growth rate
0.2
0.1
0.0
# of days after an introduction of primary cases

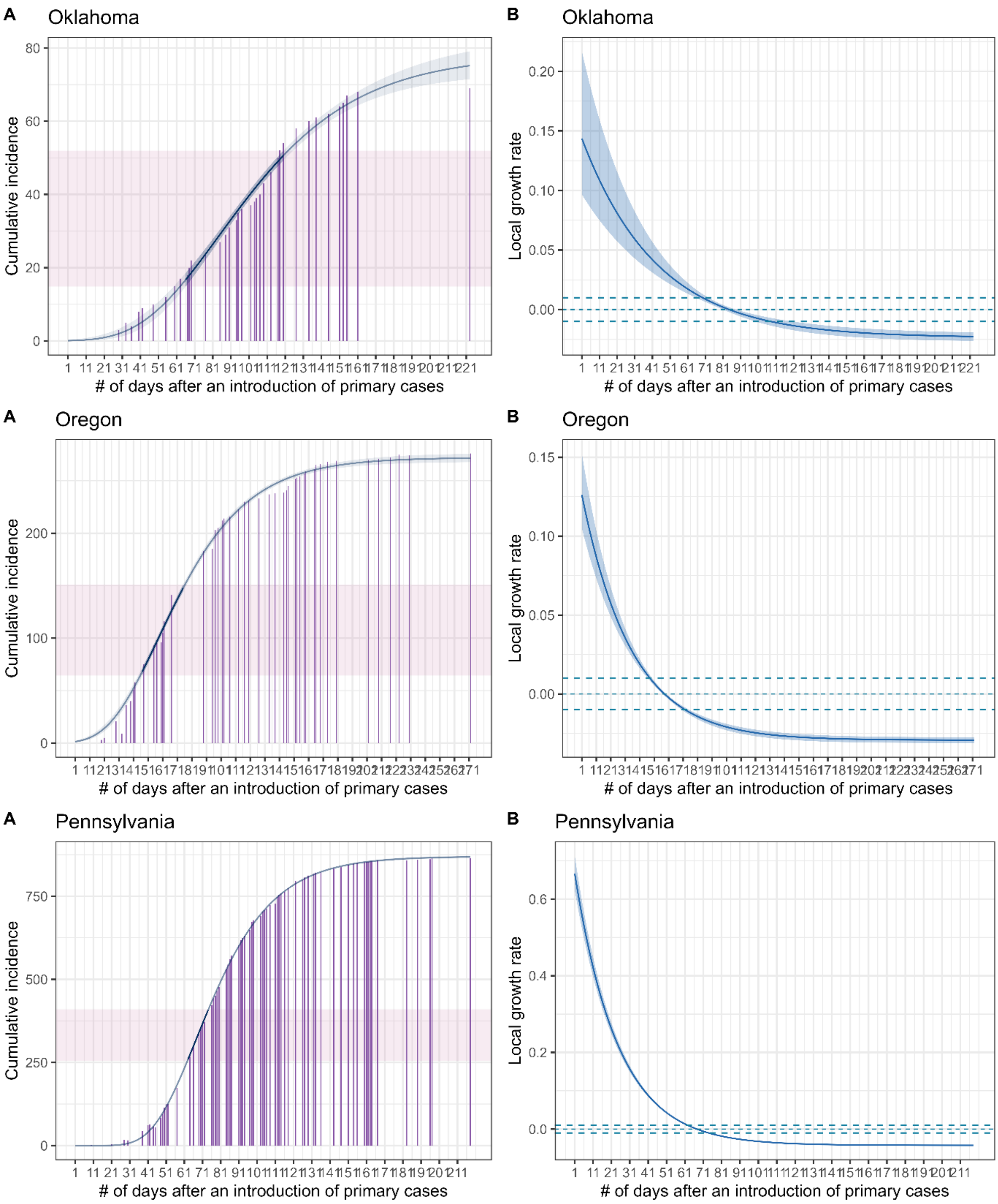
A
Oklahoma
Cumulative incidence
0
20
40
60
80
# of days after an introduction of primary cases
B
Oklahoma
Local growth rate
0.00
0.05
0.10
0.15
0.20
# of days after an introduction of primary cases
A
Oregon
Cumulative incidence
0
100
200
# of days after an introduction of primary cases
B
Oregon
Local growth rate
0.00
0.05
0.10
0.15
# of days after an introduction of primary cases
A
Pennsylvania
Cumulative incidence
0
250
500
750
# of days after an introduction of primary cases
B
Pennsylvania
Local growth rate
0.0
0.2
0.4
0.6
# of days after an introduction of primary cases

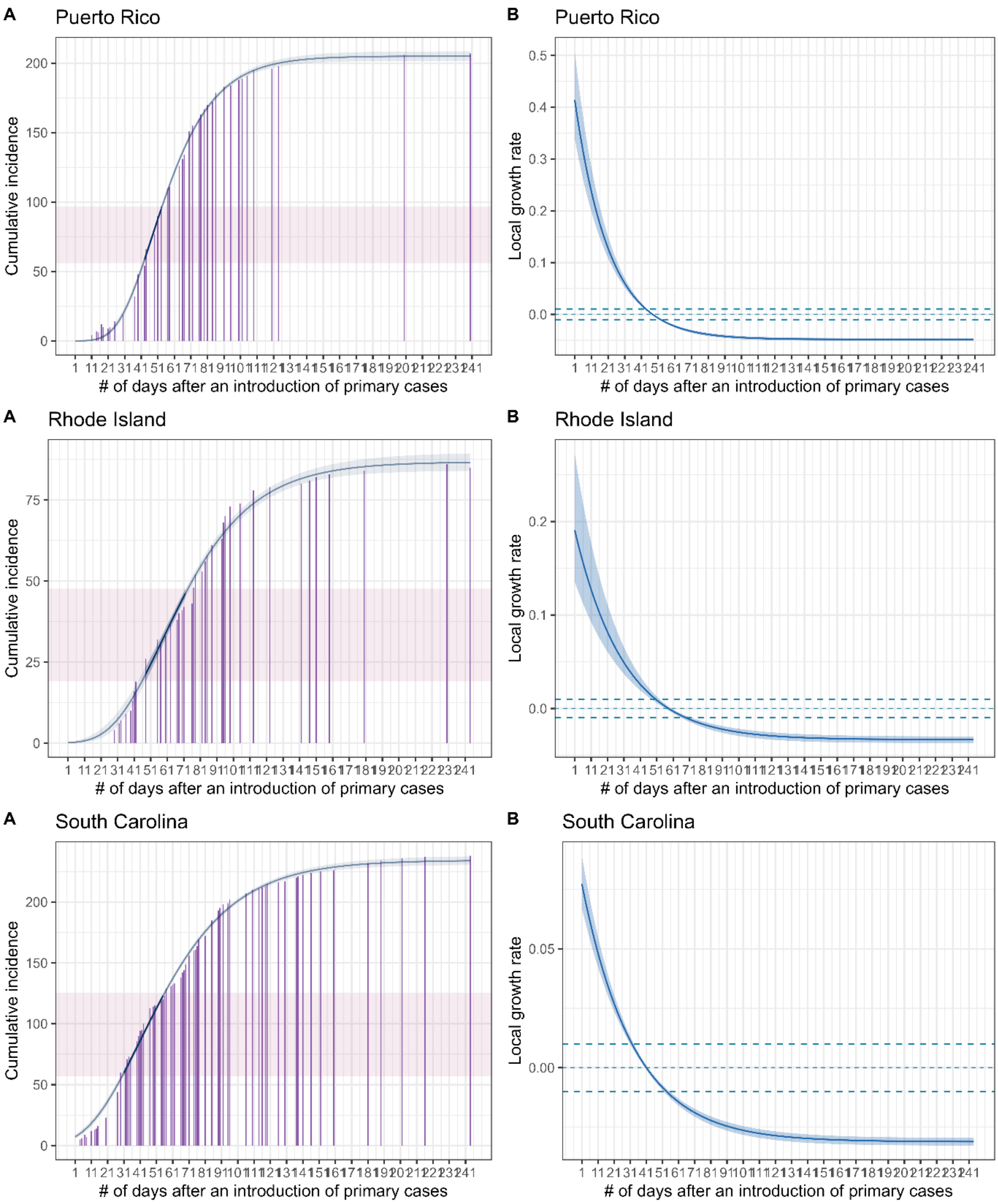
A
Puerto Rico
Cumulative incidence
0
50
100
150
200
# of days after an introduction of primary cases
B
Puerto Rico
Local growth rate
0.0
0.1
0.2
0.3
0.4
0.5
# of days after an introduction of primary cases
A
Rhode Island
Cumulative incidence
0
25
50
75
# of days after an introduction of primary cases
B
Rhode Island
Local growth rate
0.0
0.1
0.2
# of days after an introduction of primary cases
A
South Carolina
Cumulative incidence
0
50
100
150
200
# of days after an introduction of primary cases
B
South Carolina
Local growth rate
0.00
0.05
# of days after an introduction of primary cases

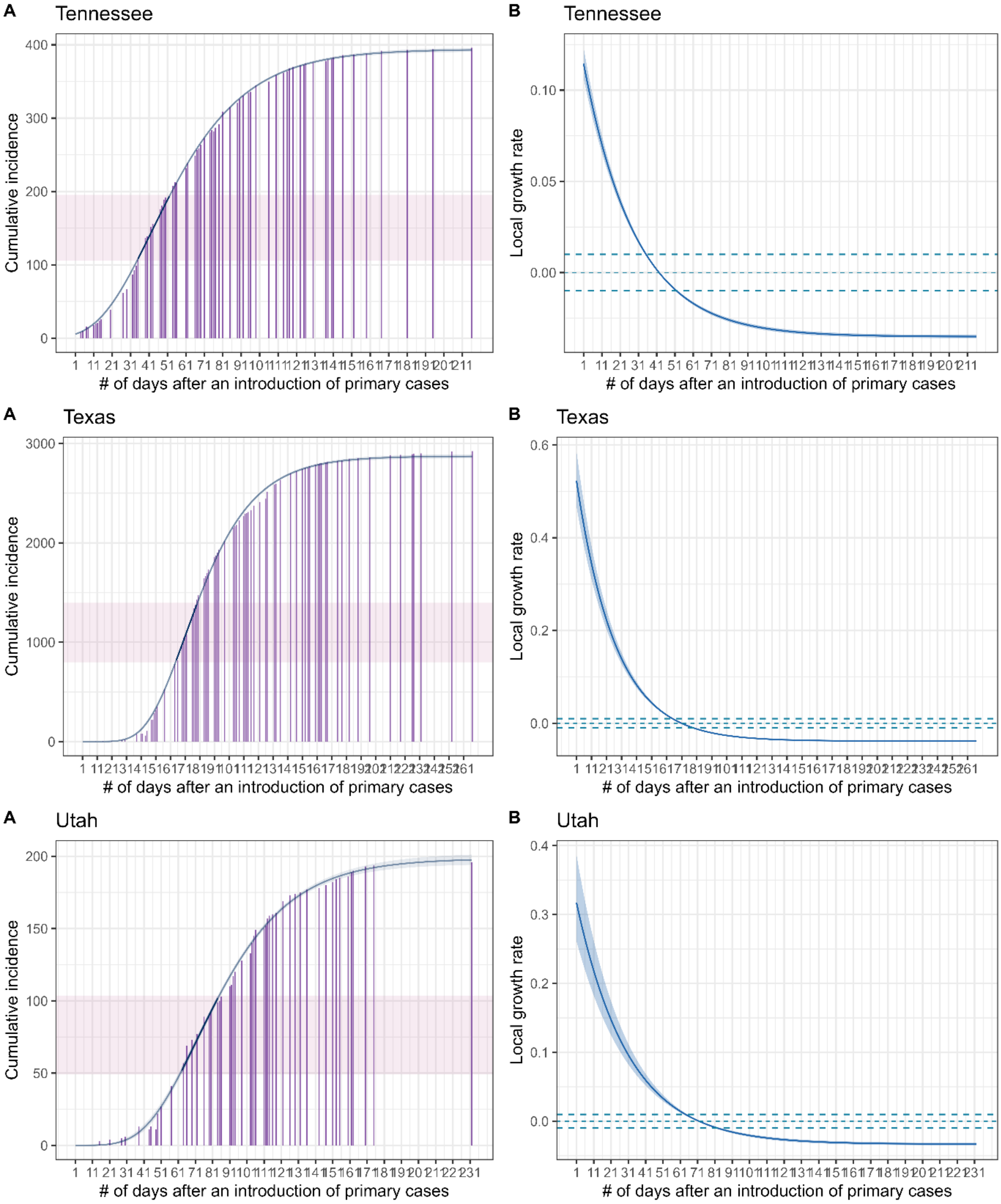
A
Tennessee
Cumulative incidence
400
300
200
100
0
# of days after an introduction of primary cases
B
Tennessee
Local growth rate
0.10
0.05
0.00
# of days after an introduction of primary cases
A
Texas
Cumulative incidence
3000
2000
1000
0
# of days after an introduction of primary cases
B
Texas
Local growth rate
0.6
0.4
0.2
0.0
# of days after an introduction of primary cases
A
Utah
Cumulative incidence
200
150
100
50
0
# of days after an introduction of primary cases
B
Utah
Local growth rate
0.4
0.3
0.2
0.1
0.0
# of days after an introduction of primary cases

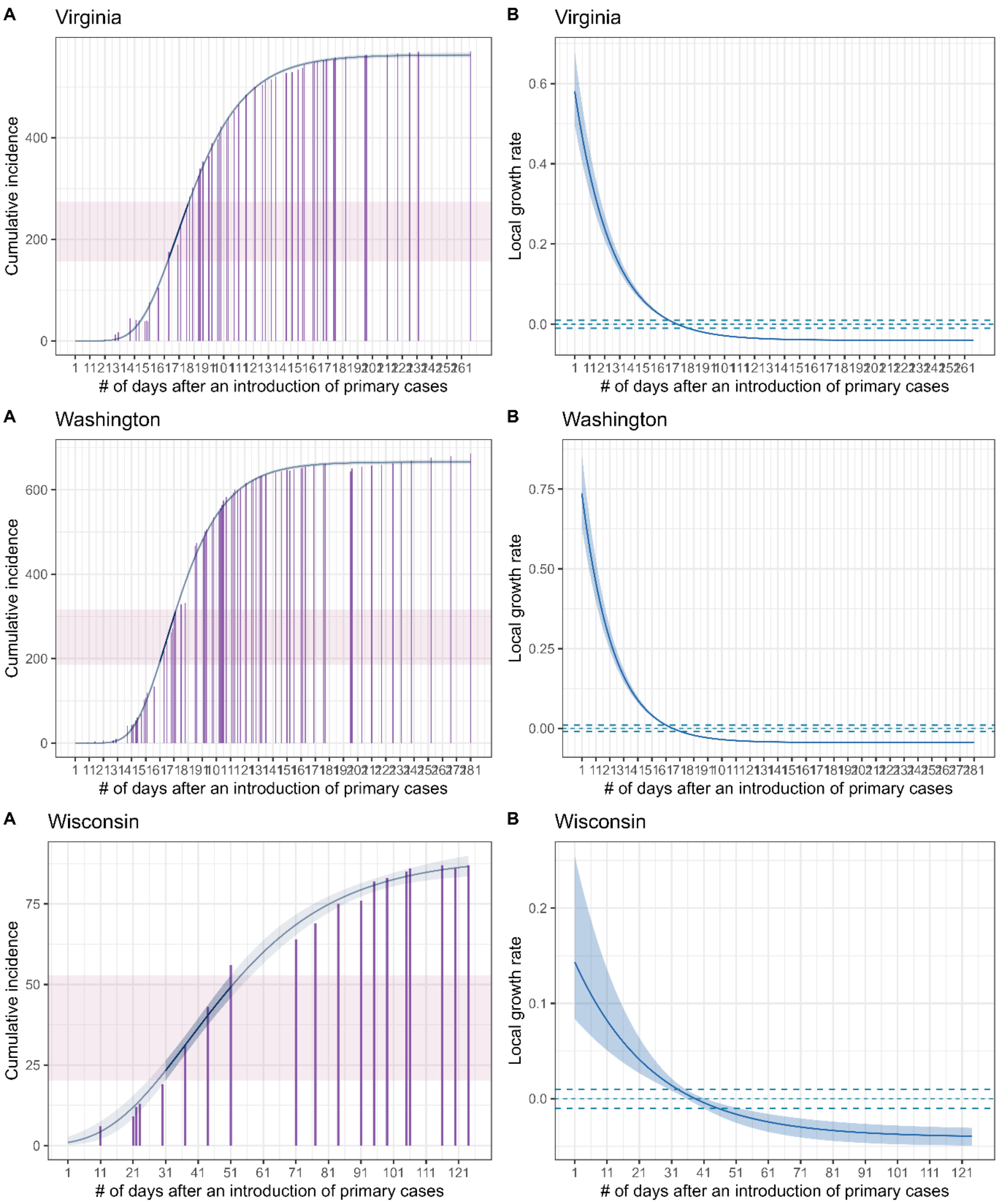

Supplementary references

1. Global.health. Monkeypox 2022 global epidemiology; Report 2022-09-23 [Internet]. 2022 [cited 2022 Nov 10]. Available from: https://www.monkeypox.global.health/
2. Centers for Disease Control and Prevention. 2022 Outbreak Cases and Data [Internet]. 2022 [cited 2022 Oct 18]. Available from: https://www.cdc.gov/poxvirus/monkeypox/response/2022/index.html
3. DeWitt Mi. Tracking Monkeypox Virus (MPX) infections reported in the United States [Internet]. 2022 [cited 2022 Oct 18]. Available from: https://github.com/wf-id/us-mpx
4. Wayback Machine. Internet Archive Wayback Machine [Internet]. [cited 2022 Nov 10]. Available from: http://web.archive.org/

5. Marcus U, Hickson F, Weatherburn P, Schmidt AJ, Network the E. Estimating the size of the MSM populations for 38 European countries by calculating the survey-surveillance discrepancies (SSD) between self-reported new HIV diagnoses from the European MSM internet survey (EMIS) and surveillance-reported HIV diagnoses among MSM in 2009. BMC Public Health [Internet]. 2013;13(1):919. Available from: https://doi.org/10.1186/1471-2458-13-919
6. Grey JA, Bernstein KT, Sullivan PS, Purcell DW, Chesson HW, Gift TL, et al. Estimating the Population Sizes of Men Who Have Sex With Men in US States and Counties Using Data From the American Community Survey. JMIR Public Health Surveill [Internet]. 2016;2(1):e14. Available from: http://publichealth.jmir.org/2016/1/e14/
7. United Nations. World Population Prospects 2022 [Internet]. 2022 [cited 2022 Oct 18]. Available from: https://population.un.org/wpp/
8. The Census Bureau. Data [Internet]. [cited 2022 Oct 18]. Available from: https://www.census.gov/data.html
9. UNAIDS. UNAIDS - KEY POPULATIONS ATLAS [Internet]. 2022 [cited 2023 Apr 16]. Available from: https://kpatlas.unaids.org/dashboard
10. UNAIDS. Key Populations Atlas Data sources [Internet]. 2017 [cited 2023 May 16]. Available from: https://kpatlas.unaids.org/document/kp_data_sources.pdf
11. Centers for Disease Prevention and Control. Monkeypox Vaccine Administration in the U.S. [Internet]. 2022 [cited 2022 Oct 18]. Available from: https://www.cdc.gov/poxvirus/monkeypox/response/2022/vaccines_data.html
12. Bürger R, Chowell G, Lara-Dıíaz LY. Comparative analysis of phenomenological growth models applied to epidemic outbreaks. Mathematical Biosciences and Engineering. 2019;16(5):4250–73.
13. Català M, Alonso S, Alvarez-Lacalle E, López D, Cardona PJ, Prats C. Empirical model for short-time prediction of COVID-19 spreading. PLoS Comput Biol [Internet]. 2020 Dec 9;16(12):e1008431-. Available from: https://doi.org/10.1371/journal.pcbi.1008431
14. Carpenter B, Gelman A, Hoffman MD, Lee D, Goodrich B, Betancourt M, et al. Stan: A Probabilistic Programming Language. J Stat Softw [Internet]. 2017 Jan 11;76(1):1–32. Available from: https://www.jstatsoft.org/index.php/jss/article/view/v076i01
15. Endo A, Murayama H, Abbott S, Ratnayake R, Pearson CAB, Edmunds WJ, et al. Heavy-tailed sexual contact networks and monkeypox epidemiology in the global outbreak, 2022. Science (1979). 2022;eadd4507.
16. Søren Asmussen. Steady-State Properties of of GI/G/1. In: Asmussen S, editor. Applied Probability and Queues [Internet]. New York, NY: Springer New York; 2003. p. 266–301. Available from: https://doi.org/10.1007/0-387-21525-5_10
17. UK Health Security Ageny. Investigation into monkeypox outbreak in England: technical briefing 1 [Internet]. UK Health Security Agency. 2022 [cited 2022 Oct 18]. Available from: https://www.gov.uk/government/publications/monkeypox-outbreak-technical-briefings/investigation-into-monkeypox-outbreak-in-england-technical-briefing-1
18. Suñer C, Ubals M, Tarín-Vicente EJ, Mendoza A, Alemany A, Hernández-Rodríguez Á, et al. Viral Dynamics in Patients with Monkeypox Infection: A Prospective Cohort Study in Spain. 2022;
19. World Health Organization. 2022 Monkeypox Outbreak: Global Trends [Internet]. 2022 [cited 2022 Oct 18]. Available from: https://worldhealthorg.shinyapps.io/mpx_global/
20. Brauer F, van den Driessche P, Wu J, Allen LJS. Mathematical epidemiology. Vol. 1945. Springer; 2008.
21. Brosius I, Van Dijck C, Coppens J, Vandenhove L, Bangwen E, Vanroye F, et al. Pre- and asymptomatic viral shedding in high-risk contacts of monkeypox cases: a prospective cohort study. medRxiv. 2022;2022.11.23.22282505.
22. Badham J, Stocker R. The impact of network clustering and assortativity on epidemic behaviour. Theor Popul Biol. 2010;77(1):71–5.
